# A Compact Ultra-Wideband Vivaldi Antenna for Short-Range Vehicular Communications Using Radial-to-Radial Transition and Matching Transformer

**DOI:** 10.3390/s26206660

**Published:** 2026-10-21

**Authors:** Sahar Saleh, Tale Saeidi, Faroq Razzaz, Nick Timmons, Ayman A. Althuwayb, Bader Alali, Qusay Shihab Hamad, Dokhyl AlQahtani, Saud bin Saeed

**Affiliations:** 1 WiSAR Lab, Atlantic Technological University (ATU), F92 YY97 Letterkenny, Co. Donegal, Ireland; tale.saeidi@atu.ie (T.S.); nick.timmons@atu.ie (N.T.); 2 Department of Electronic Engineering and Communications, Faculty of Engineering, University of Aden, Aden P.O. Box 5243, Yemen; 3 Electrical and Electronics Engineering Department, Faculty of Engineering and Natural, Sciences, Istinye University, Istanbul 34326, Turkey; 4 Electrical Engineering Department, Prince Sattam Bin Abdulaziz University, Al-Kharj 16273, Saudi Arabia; f.kasim@psau.edu.sa (F.R.); dm.alqahtani@psau.edu.sa (D.A.); 5 Faculty of Engineering and Information Technology, Taiz University, Taiz 6803, Yemen; 6 Electrical Engineering Department, College of Engineering, Jouf University, Sakaka 72388, Saudi Arabia; aaalthuwayb@ju.edu.sa (A.A.A.); bsalali@ju.edu.sa (B.A.); 7 University of Information Technology and Communications (UoITC), Baghdad 10001, Iraq; qusay.phd@gmail.com; 8 Prince Sultan Defense Studies and Research Center (PSDSARC), Riyadh 13322, Saudi Arabia; saud.binsaeed@psdsarc.org.sa

**Keywords:** ultra-wide band (UWB), vehicular communication, Vivaldi tapered slot antennas (VTSA), radial to radial microstrip to slot (M/S) transition, matching transformer and CST

## Abstract

This work presents a compact ultra-wideband (UWB) Vivaldi tapered slot antenna for short-range vehicular communication and broadband UWB applications. The proposed design is developed using two simple performance-enhancement and size-reduction techniques: a radial-to-radial microstrip-to-slot transition and an impedance-matching transformer. The antenna employing only the radial-to-radial transition is referred to as VTSAR, while the final design incorporating both the radial-to-radial transition and the matching transformer is referred to as VTSARMT. Unlike many compact UWB antenna designs that rely on complex structural loading, parasitic elements, or additional decoupling components, the proposed approach improves compactness, impedance bandwidth, and gain through a simple feeding-transition modification supported by systematic parametric analysis. The intermediate VTSAR provides a 9.84% footprint-area reduction relative to the reference VTSA, while the final VTSARMT further increases the footprint-area reduction to 16.67%. The fabricated VTSARMT exhibits measured impedance matching below −10.61 dB from 2.58 to 22.34 GHz, corresponding to a 47.67% measured bandwidth improvement relative to the reference VTSA. The maximum measured realized gain increases from 6.51 dBi for the reference VTSA to 7.45 dBi for the VTSARMT, while the measured total radiation efficiency ranges from 47.7% to 94.08%. An approximately stable measured group delay of 1 ns is obtained across most of the operating band. To assess the effects of vehicle integration, the proposed antenna is further evaluated through CST-based stationary rooftop-mounted two-vehicle and three-vehicle configurations. The analysis includes radiation-pattern assessment, transmission coefficients, and CST-based received-power evaluation. These simulations provide a comparative assessment of stationary directional links rather than experimental validation of a dynamic V2V channel. The CST-based vehicle analysis provides a preliminary assessment of rooftop integration and stationary directional V2V links under controlled simulation conditions.

## 1. Introduction

Vehicular communication has become a key enabling technology for intelligent transportation systems (ITS), connected vehicles, and future autonomous mobility. Modern vehicles are increasingly expected to exchange information with surrounding vehicles, roadside infrastructure, pedestrians, networks, and satellites through vehicle-to-everything (V2X) communication links, including vehicle-to-vehicle (V2V), vehicle-to-infrastructure (V2I), vehicle-to-pedestrian (V2P), vehicle-to-network (V2N), and vehicle-to-satellite (V2S) communications [1,2,3,4]. These links are essential for improving road safety, traffic efficiency, emergency response, cooperative driving, and real-time situational awareness [2]. Recent vehicular antenna research has therefore focused on designs that can provide wide bandwidth, compact size, stable radiation characteristics, high gain, and reliable performance under high-mobility and multipath-rich propagation conditions [4,5,6,7]. The recent proposed V2X antenna studies have emphasized the necessity of antennas capable of handling multiple vehicular communication links and frequency bands, such as compact MIMO, switched-beam, wideband reflectarray and phased-array antenna solutions [4,5,6,7].

Ultra-wideband (UWB) wireless communication is an innovative technology that provides remarkable opportunities in the current wireless communication system because of its unique structures and features, such as low cost, high data rate, and small physical dimensions, in addition to low power consumption. In addition, the unique characteristics of ultra-wideband technology make it more suitable for use in medical applications, military security, and civilian commerce [8].

The Exponentially or Vivaldi Tapered Slot Antenna (ETSA or VTSA) is the optimal choice to fulfill the requirements of UWB technology due to its unique end-fire symmetrical radiation pattern, wide bandwidth (BW), simple design, low-profile, low-cost manufacturing, low power consumption, and easy integration with other circuits. Several techniques have been proposed in the literature to improve the performance of Vivaldi antennas and to reduce their dimensions and size for different applications [9].

Based on the concept of increasing the electrical length to reduce the ETSA size, researchers in both [10,11] removed its conducting edges to design a compact wideband (6–9 GHz) and UWB (6–18 GHz) Double ETSAs (DETSAs), respectively. However, in [12,13], a pair of tapered slot edges (TSE) and a symmetrical resonant structure are added to the radiation patches to lower the size of the broadband (2.6–20 GHz) TSA and wideband (2.7–2.9 GHz) VTSA, respectively.

In addition to using corrugations to reduce the size of UWB (3.55 to more than 12 GHz) VTSA [14] and UWB (2.92–11.91 GHz) binomial transformer TSA (BTSA) [15], they are exploited to enhance their BW by 55.85% and 1.89% (Simulated (Sim.)), respectively.

In [16], to achieve the essential requirements for Ground Penetrating Radar (GPR) and See-Through Wall (STW) applications in terms of narrow beam, high resolution, and depth penetration (1–1.15 m), four pairs of circular cavities and very thin slots are etched in the UWB (1.17–4.75 GHz) VTSA’s radiator patch. In [17], the use of a continuous sawtooth outer edge and two pairs of inclined elliptical slots enhances the gain by 2.8 dBi and expands the bandwidth, making it ideal for applications such as through-wall radar, microwave imaging, and wireless communication. In [18] fan-shaped flare and stepped slot structures are used in the proposed VTSA, resulting in a 45.9% compactness, a 142.6% impedance BW, and a high gain of 7.9 dBi, suitable for UWB through-wall radar and breast cancer detection applications. Authors in [19] further improved the antipodal Vivaldi antenna by using a Klopfenstein taper profile combined with slotting, director loading, and perforation techniques, achieving a measured wide BW of 2–20 GHz and a maximum gain of 14 dBi.

The resonant frequency can be adjusted and reduced for compactness by employing short pins and resistors during the feeding transition, as demonstrated in [20]. The tapered slot profile or microstrip-to-slot (M/S) transition of the antenna can be altered to achieve compactness. For example, reducing the entire Vivaldi antenna to half its original size (Monopole) [21], modifying the (M/S) transition from rectangular to circular [8], and applying Vivaldi non-uniform slot profile antenna (VNSPA) theory [22] can lead to size reductions of 77.51%, 19.25%, and 51.94%, respectively. In [23], the VNSPA theory is exploited to achieve further compactness up to 72.74% and 81.8% compared to the VTSA in [8].

The antenna directive properties can be enhanced using grating elements (lenses, metamaterials (MTM), or metals) [24,25]. In [26], three metal patches are loaded onto the (2–18.7 GHz) VTSA’s aperture to improve its directive properties. However, in [27] dielectric sheets (DS) are used to enhance the gain and reduce the half-power beamwidth (HPBW) and side lobe levels (SLLs) of the proposed Conventional Vivaldi Antenna (DSCVA). Gain and matching of the CVA in [28] are enhanced using an artificial materials lens (AML) besides a sidelobe suppressor (SSR). Similarly, authors in [29] enhanced the directivity of an antipodal Vivaldi antenna by introducing slits, an elliptical dielectric lens, and an SRR-based metasurface, achieving stable radiation characteristics and gain of 8.5–13 dBi over 9–41 GHz. Extra-high gain and wide BW can be achieved using both corrugations and gratings as in [30,31]. However, they are used in [32,33] for size reduction and performance enhancement.

To improve UWB VTSA’s far-field applications (gain and radiation patterns), MTMs and rectangular slotted patches with four star-shaped parasitic components have been added to the (2.0–10.45 GHz) VTSA in [34] and (3.8–10.1) TSA in [35], respectively, proposed for BCD. More recently, ref. [36] proposed a high-gain (15.84 dBi) miniaturized (2–20 GHz) antipodal Vivaldi antenna using tapered exponential edge slots and an embedded rhombus-shaped metamaterial array.

In [37], the UWB detection performance is improved in the proposed VTSA (3–8 GHz) by etching bottle-shaped slots on both sides of the antenna and including rectangular metal strips in the aperture. The alterations enhance the surface current path and localize the electromagnetic energy, thereby enhancing the radiation efficiency and gain. The design provides better UWB performance and is suitable for applications such as detection systems, where high resolution and better radiation characteristics are required. The proposed TSA in [38] uses a loaded dielectric lens, which improves the impedance BW (0.6–20 GHz) and gain (4.7 dBi) in the frequency range of 12–20 GHz. Optimizing the slot line curve and elliptical transition enhanced the performance, making the design suitable for UWB applications including spectrum sensing and direction-finding systems.

UWB technology is attractive for vehicular communication because of its wide impedance bandwidth, low power transmission, high data-rate capability, and robustness against multipath propagation [39,40]. These features are highly relevant in automotive environments, where signals are affected by metallic vehicle bodies, moving obstacles, passengers, road infrastructure, and rapidly changing channel conditions [39]. Consequently, UWB has also been investigated for in-car communications, high-speed mobile links, automotive localisation and tracking, roadside assistance, and emergency services. A compact UWB automotive antenna covering 3.1–10.9 GHz was previously shown to be suitable for shark-fin housing or integration with existing PCB electronics, demonstrating the practical relevance of UWB antennas for modern automotive platforms [39].

In addition to communication, UWB is also a promising technology for vehicular positioning and cooperative localisation. In GNSS-challenged or GNSS-denied urban scenarios, UWB ranging can support V2V and V2I cooperative positioning by providing accurate inter-vehicle and V2I distance measurements. Experiments on four vehicles showed that UWB ranging can achieve relative positioning performance at the sub-meter level, especially when fused with vision data or when there are enough V2V ranging links [40]. This makes UWB especially relevant for intelligent transportation applications where communication and localisation are both required for collision avoidance, platooning, cooperative perception, and autonomous navigation.

Despite these advantages, the practical integration of UWB antennas into vehicles remains challenging. Many reported UWB and vehicular antennas achieve wide bandwidth and enhanced performance through large footprints, complex loading structures, decoupling networks, or additional radiating elements, which can increase fabrication complexity and complicate vehicle integration. Consequently, there remains a need for compact, broadband, and structurally simple antenna solutions capable of supporting realistic vehicular communication and localisation applications [39]. MIMO and diversity antennas are widely used to improve link robustness, reduce fading effects, increase channel capacity, and maintain communication reliability in multipath vehicular environments. However, many existing UWB and vehicular MIMO antennas use complex decoupling structures, large footprints, or additional loading elements, which can complicate fabrication and make practical automotive integration difficult [39].

Various antenna technologies have been proposed for V2X communication. The importance of compact planar antennas for V2V, V2I, and V2N applications is illustrated by developments of compact CPW-fed MIMO antennas for 3.5 GHz LTE-42 and 5.9 GHz DSRC bands [41]. A compact octagonal UWB patch antenna is proposed in [42] for V2X/DSRC and multi-standard wireless applications covering 4.2–8.9 GHz with a measured bandwidth of 4.7 GHz and supporting DSRC/IEEE 802.11p, WiMAX, WLAN, Wi-Fi 6/6E, 5G sub-6 GHz, and early 6G-related bands.

Authors in [2] proposed a switched-beam V2V communication framework incorporating a low-complexity beam-management algorithm to improve link reliability under different vehicular environments. A capacitively fed low-profile UWB (0.75–7.6 GHz) monocone antenna with folded shorting wires was proposed in [43] for V2X applications and demonstrated improved communication range through vehicle-based field testing using an onboard unit (OBU) and roadside unit (RSU). The study in [44] presented a compact hexagonal ring-inspired UWB antenna covering 3.33–19.94 GHz with a WLAN notch from 5.04–6.01 GHz and stable radiation performance.

A compact reconfigurable MIMO antenna framework was proposed in [7] for V2X communication, integrating defected ground structures (DGSs), parasitic elements, PIN diodes, adaptive control, and optimisation techniques to achieve high efficiency and low correlation. Although strong antenna and diversity performance is reported, including ECC below 0.02, gain up to 7.65 dBi, and efficiency up to 93%, the validation is mainly based on antenna-level measurements and synthetic-data-driven adaptive modelling. The description does not explicitly show a realistic vehicle-mounted or inter-vehicle EM link scenario, which underlines the need for further on-vehicle and multi-vehicle assessment. The work in [3] developed a compact multiband shark-fin antenna for integrated V2X communication systems supporting FM (88–108 MHz), TETRA (380–470 MHz), cellular, and Wi-Fi services within a rooftop-compatible structure. Authors in [6] presented a compact four-channel MIMO vehicle antenna supporting 5G sub-6 GHz, LTE, WLAN, and V2X communications. A circularly polarised wideband high-gain reflectarray antenna for future V2X systems was reported in [4], providing 12–32 GHz gain bandwidth for V2S, V2V radar, and mmWave communication links. Although these studies demonstrate significant progress in vehicular antenna design, most focus primarily on antenna characteristics, diversity performance, communication algorithms, or system-level metrics. Detailed electromagnetic assessment under realistic rooftop-mounted and multi-vehicle scenarios remains relatively limited, particularly for transmission-coefficient-based V2V link evaluation. This motivates the need for practical vehicle-mounted investigations incorporating surrounding-vehicle effects and inter-vehicle link analysis, which forms a key objective of the present work.

The abovementioned characteristics of VTSAs make them suitable for vehicular UWB applications, where compact integration, directional coverage, and stable broadband performance are required in the presence of vehicle-body effects and dynamic propagation conditions. Vivaldi antennas have already been used in V2X measurement systems; for example, a dual-polarised Vivaldi antenna pair operating from 560 MHz to 7.7 GHz was reported in [45] for V2X communication measurement, covering existing V2X communication frequencies with high isolation and useful gain. Ref. [46] reported an integrated three-port Vivaldi diversity antenna for UWB vehicular applications, achieving three directional radiation patterns, a 5–11 GHz impedance bandwidth, port-to-port isolation greater than 20 dB, and measured gain from 2.3 to 6.6 dB. A compact multiband (3.5, 3.8, 5.7, and 7.4 GHz) two-element Vivaldi antenna array was proposed in [47] for autonomous vehicle V2X communication. Side slots and a triangular director are used to achieve multiband operation and a high gain of 5.3–8 dBi. The work includes CST simulations, prototype measurements, and outdoor RSSI/SNR comparisons, demonstrating the suitability of the antenna for vehicular communication applications. Authors in [48] developed an omnidirectional low-profile dual-polarised UWB antenna for IoV applications, in which a circular array of eight dual-antipodal Vivaldi antennas was used to realise the horizontally polarised element, while crossed tapered monopoles provided the vertically polarised element. The antenna covered 3.06–10.62 GHz and was intended for Passive Keyless Entry and Start (PKES) and Automated Valet Parking (AVP) applications rather than an explicit inter-vehicle V2V link scenario. These studies demonstrate the use of Vivaldi antennas in selected vehicular applications; however, detailed rooftop-mounted and multi-vehicle V2V link investigations remain limited in the current literature. Although omnidirectional antennas are advantageous for continuous azimuthal coverage, the end-fire Vivaldi antenna concentrates radiated energy towards a selected direction and can therefore be considered for orientation-controlled front-, rear-, or side-directed vehicular links. Accordingly, the present study considers simplified stationary, orientation-controlled directional links rather than complete $360^{\circ }$ azimuthal coverage. Wider angular coverage could potentially be achieved using multiple differently oriented Vivaldi elements or diversity/MIMO configurations, as demonstrated by previously reported multi-element Vivaldi approaches for vehicular applications [47].

In our previous work, a compact 5G mmWave Vivaldi antenna was developed for vehicular communication using Vivaldi Non-uniform Slot Profile Antenna theory [1]. That work demonstrated that compact Vivaldi antennas can provide wide bandwidth, moderate-to-high gain, and practical vehicle-mounted radiation behaviour in the 26 GHz band. It also investigated on-vehicle performance and SAR effects for drivers, passengers, and pedestrians, confirming the importance of evaluating vehicular antenna designs in realistic automotive scenarios [1]. Building on this motivation, the present work shifts the focus from mmWave operation to the UWB frequency range, where the antenna can support wideband vehicular communication, short-range V2X links, cooperative localisation, and automotive sensing-related applications.

From the reviewed vehicular antenna studies, significant progress has been achieved in compact V2X antennas, UWB automotive antennas, MIMO/diversity structures, and Vivaldi-based vehicular antennas. However, many reported studies focus primarily on antenna-level performance, while detailed vehicle-mounted and multi-vehicle electromagnetic assessments remain comparatively limited. In addition, despite substantial advances in Vivaldi antenna miniaturisation and performance enhancement, simultaneously achieving compact size, wide bandwidth, sufficient gain, stable radiation behaviour, and low structural complexity remains challenging for vehicular integration.

To address these aspects, the present work experimentally evaluates the proposed antenna and subsequently investigates its rooftop integration using simplified stationary, orientation-controlled two-vehicle and three-vehicle CST configurations. The vehicle analysis examines the effects of rooftop mounting and surrounding vehicle bodies on antenna matching, realized gain, radiation characteristics, transmission coefficients, and received power. These simulations are intended as a comparative assessment of stationary directional links rather than full-scale dynamic V2V-channel validation.

Vivaldi antennas are inherently broadband, and very wide fractional bandwidths have been demonstrated using conventional and modified tapered-slot configurations. Tapered, stepped, and multi-section microstrip feeding structures are also established techniques for improving broadband impedance matching [49]. Therefore, neither the wideband nature of the Vivaldi antenna nor the individual use of a matching transformer is claimed as novel in this work.

Against this background, the specific contribution of this work lies in the combined implementation and full-wave optimisation of a radial-to-radial microstrip-to-slotline transition and a quarter-wavelength matching transformer within the compact VTSA architecture reported in [8]. The reference VTSA employs a circular-to-circular microstrip-to-slotline transition without a matching transformer. In the proposed configuration, the radial-to-radial transition modifies the electromagnetic coupling between the microstrip feed and the tapered slot, while the optimised quarter-wavelength transformer provides impedance transformation between the 50 Ω input line and the radial feeding section. The resulting VTSA with radial-to-radial transition and matching transformer (VTSARMT) maintains a simple single-layer structure without requiring lenses, metamaterials, directors, parasitic elements, or multilayer structures.

The proposed VTSARMT is fabricated and experimentally characterised in terms of impedance matching, realized gain, radiation patterns, efficiency, phase, and group delay. Its rooftop integration is further assessed through the stationary two-vehicle and three-vehicle CST configurations described above. Thus, the contribution of this work lies in the combined antenna design and optimization, experimental validation, and preliminary stationary vehicular assessment of the compact VTSARMT, rather than in claiming either feeding technique or ultra-wideband Vivaldi operation as independently novel.

The remainder of this paper is organized as follows: Section 2 presents the antenna design, optimization, and electromagnetic analysis. Section 3 evaluates the on-vehicle integration and stationary directional links. Section 4 presents the simulated and measured antenna results. Finally, Section 5 concludes the work.

## 2. Design and Analysis

**Figure 1:**
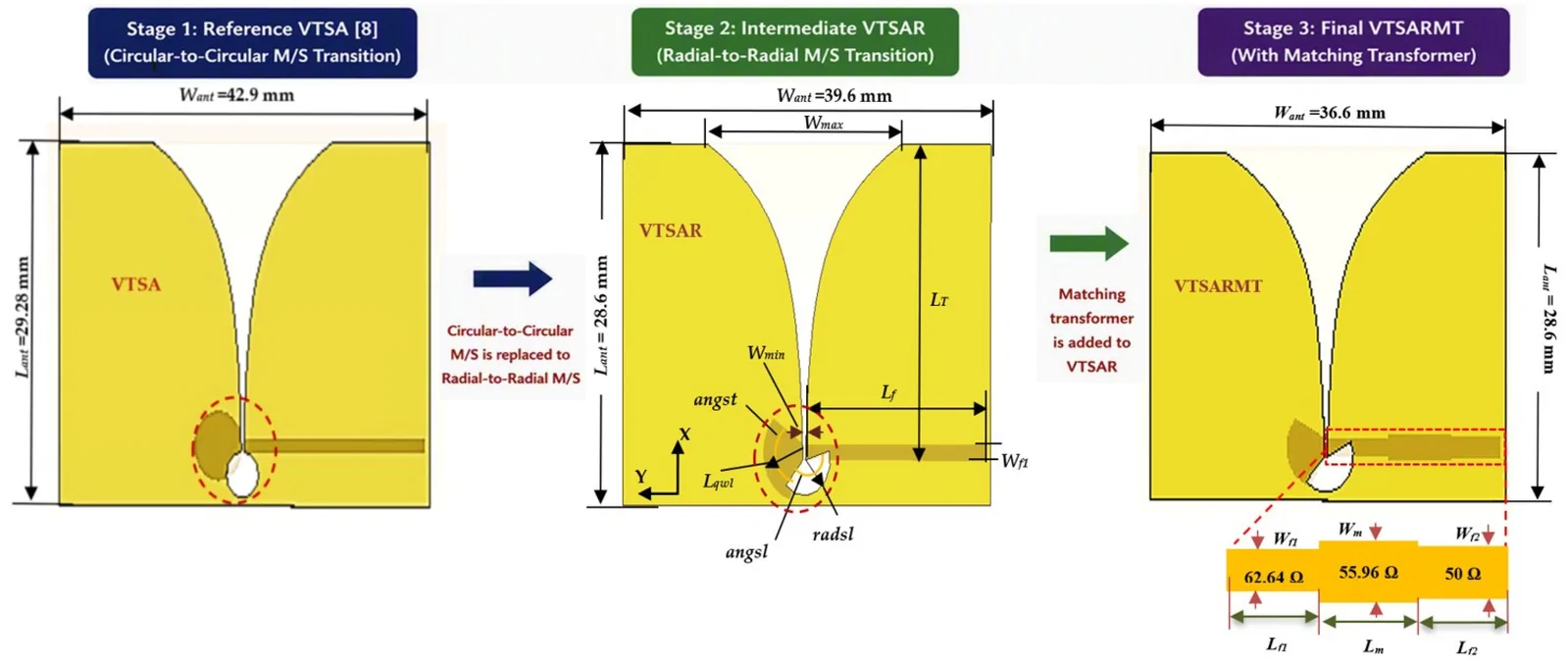
Design evolution of the proposed compact UWB VTSARMT antenna from the reference VTSA through the VTSAR and VTSARMT configurations.

The proposed VTSARMT was developed from the compact UWB VTSA reported in [8]. The reference VTSA employs a circular-to-circular microstrip-to-slotline transition without a matching transformer. In the intermediate design, the circular-to-circular transition is replaced by a radial-to-radial structure, which modifies the electromagnetic coupling region between the microstrip feed and the tapered slot. This configuration is referred to as the Vivaldi tapered slot antenna with radial-to-radial transition (VTSAR). The final VTSARMT (VTSAR with matching transformer) additionally incorporates an optimised quarter-wavelength transformer between the 50 Ω input line and the radial feeding section to provide impedance transformation. Therefore, the proposed configuration differs from conventional stepped-impedance matching alone by combining coupling-aperture modification with full-wave optimisation of the matching transformer within the compact VTSA structure. Figure 1 illustrates the design evolution from the reference VTSA to the intermediate VTSAR and the proposed VTSARMT. All antenna configurations were designed on a Rogers RO4003C substrate with a relative permittivity of $\varepsilon_{r}=3.55$ and a thickness of h=0.813 mm.

In Figure 1, the dimensions, $W_{min}$ and $W_{max}$ are the minimum and maximum widths of the tapered slot, respectively; $L_{qw}$ is the quarter-wavelength slot length; radsl the slot radius; angsl and angst are the slot and stub angles, respectively; $L_{T}$ is the tapered-slot length; $L_{f}$ is the total feed length; $W_{ant}$ and $L_{ant}$ are the overall antenna width and length, respectively; and $dis=L_{ant}-L_{qw}$ represents the remaining longitudinal distance after the quarter-wavelength slot section. The parameter r denotes the exponential taper rate.

The detailed parametric optimization of $L_{qw}$, dis, radsl, angsl, angst, and $W_{f1}$ for the intermediate VTSAR was previously reported in [49]. The optimized values were $L_{qw}=4.6$ mm, dis=35 mm, $rad_{sl}=2$ mm, $ang_{sl}=150^{\circ }$, angst, and $W_{f1}=1.25$ mm, achieving $|S_{11}|\;<-11.37$ dB over 3.29–17.55 GHz with a maximum realized gain of 7.19 dBi. The taper rate, minimum slot width, and maximum aperture width were maintained at r=0.17, $W_{min}=0.3$ mm, and $W_{max}=21.03$ mm, respectively. The resulting VTSAR has a physical footprint area of $1132.56\;{mm}^{2}$, calculated as $W_{ant}\;\times \;L_{ant}=39.6\times 28.6\;{mm}^{2}$. Relative to the reference VTSA footprint area of $1256.11\;{mm}^{2}$, this corresponds to a footprint-area reduction of 9.84%. The optimized VTSAR is used as the starting configuration for investigating the additional effect of the matching transformer. As illustrated in Figure 1, for VTSARMT, a quarter-wavelength matching transformer was introduced between the 50 Ω input feed line and the optimized VTSAR feeding section. In this configuration, $W_{f2}$ and $L_{f2}$ denote the width and length of the 50 Ω input feed-line section, respectively; $W_{m}$ and $L_{m}$ denote the width and length of the quarter-wavelength matching transformer, respectively; and $W_{f1}$ and $L_{f1}$ denote the width and length of the feeding section connected to the radial stub.

The initial characteristic impedance of the quarter-wavelength transformer was calculated as

(1) $$Z_{m}=\sqrt{Z_{1}Z_{2}},$$

where $Z_{1}=50\;\Omega$ is the characteristic impedance of the input feed line and $Z_{2}=62.64\;\Omega$ is the characteristic impedance corresponding to the optimized VTSAR feeding width of $W_{f1}=1.25$ mm. This gives $Z_{m}=55.96\;\Omega$, corresponding to an initial transformer width of $W_{m}=1.51$ mm and a quarter-wavelength transformer length of $L_{m}=6.61$ mm. The calculated width of the 50 Ω input feed line was $W_{f2}=1.82$ mm. Based on the initial symmetrical division of the remaining feed length, $L_{f1}=L_{f2}=5.845$ mm.

**Figure 2:**
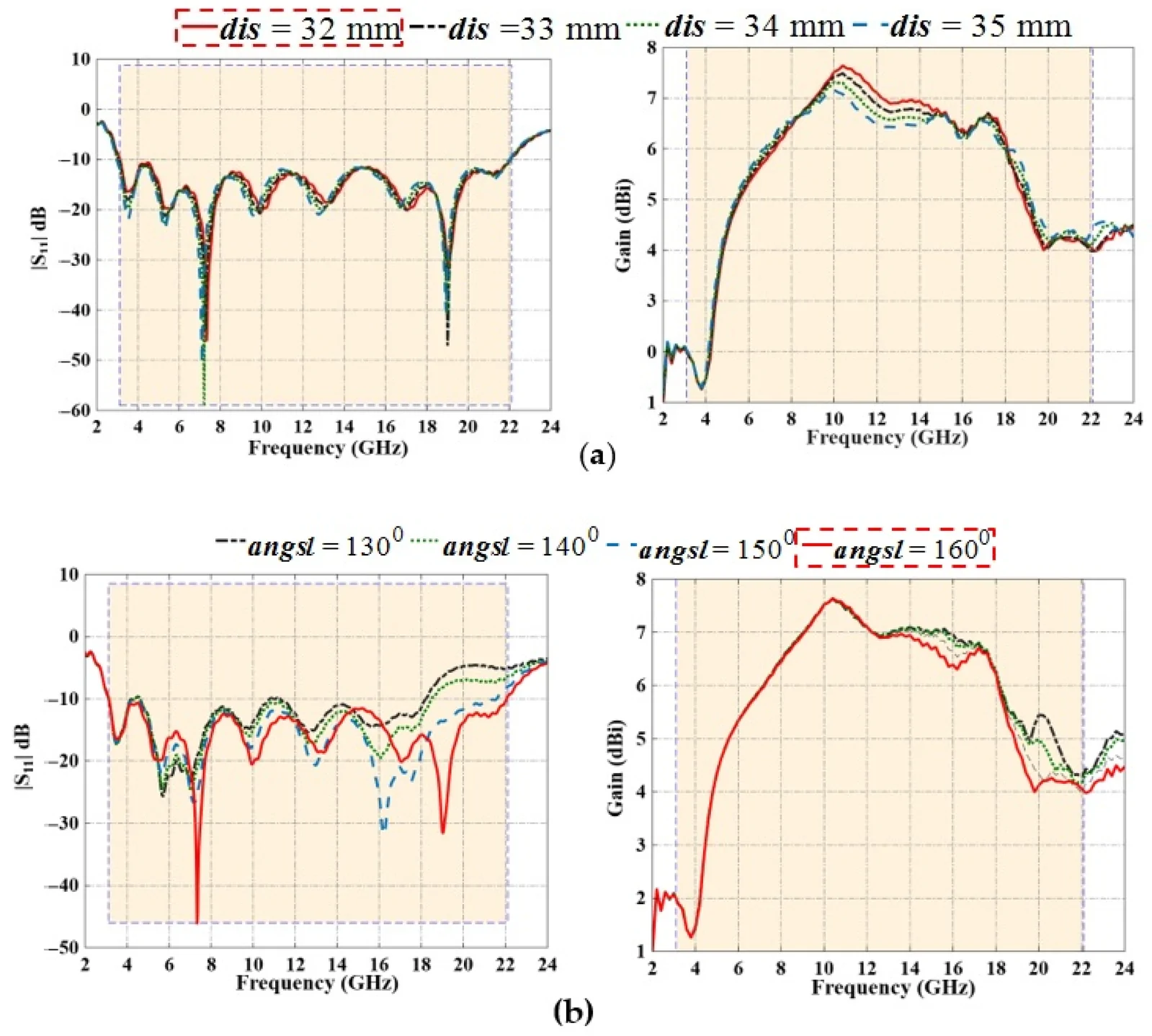

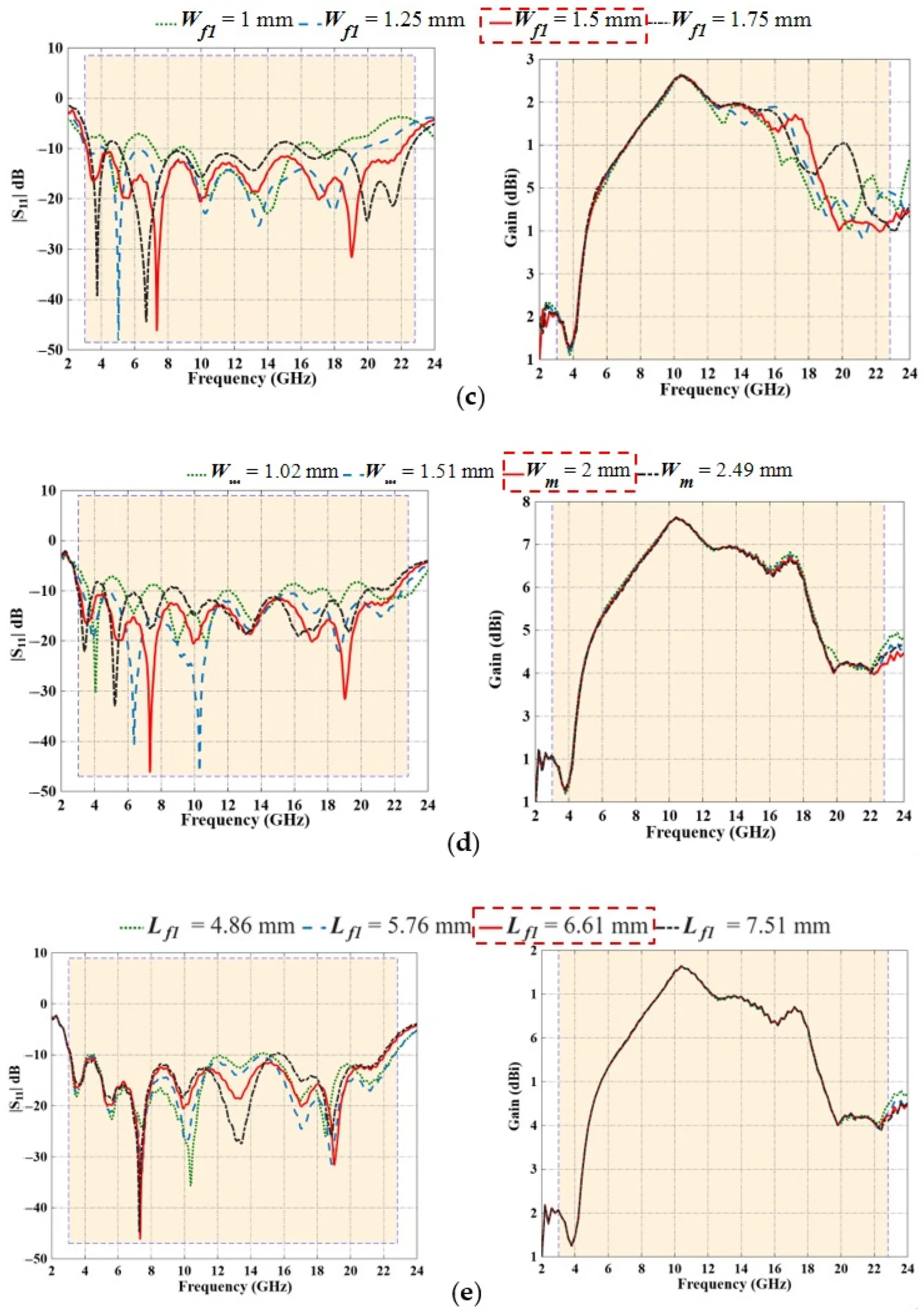
S_11_ and gain parametric study of the proposed compact UWB VTSARMT with (**a**) *dis*, (**b**) *angsl*, (**c**) *W_f_*_1_, (**d**) *W_m_*, and (**e**) *L_f_*_1_. The optimised value of each investigated parameter is indicated by a red dashed rectangle.

These analytically calculated dimensions were used as initial design values rather than as the final dimensions. The conventional transmission-line model does not fully account for the broadband discontinuity, fringing fields, and electromagnetic coupling among the input feed line, matching transformers, radial transition, and tapered slot [50]. Therefore, dis, $ang_{sl}$, $W_{f1}$, $W_{m}$, and $L_{f1}$ were subsequently investigated through full-wave parametric analysis, as shown in Figure 2. The optimised value of each investigated parameter is indicated by a red dashed rectangle in the corresponding legend in Figure 2. The input feed-line width $W_{f2}$ was fixed at 1.82 mm, corresponding to 50 Ω, while $L_{m}$ was retained at 6.61 mm. Figure 2 presents the effects of the investigated parameters on $S_{11}$ and realized gain and provides the basis for selecting the final VTSARMT dimensions. The final parameter values were selected by considering the combined effects on impedance matching across the operating band, absolute impedance bandwidth, maximum realized gain, and physical footprint. Therefore, the selected value does not necessarily provide the highest isolated gain at every frequency when another value produces a less favourable impedance response or a larger antenna footprint.

Figure 2a shows the effect of dis. Reducing dis from 35 (VTSAR) to 32 mm decreases the antenna area from 1132.56 to 1046.76 mm^2^ and increases the maximum realized gain from 7.19 to 7.64 dBi. The value dis=35 mm provides a slightly lower operating-band edge and stronger minimum matching, with $|S_{11}|<-11.64$ dB over 2.96–22 GHz. However, dis=32 mm maintains ultra-wideband matching, with $|S_{11}|<-10.64$ dB over 3.13–22.05 GHz, while providing a smaller footprint and higher maximum realized gain. Therefore, dis=32 mm was selected as the best trade-off among compactness, bandwidth, and gain. As shown in Figure 2b, varying angsl  primarily affects the impedance response, whereas the corresponding gain curves remain broadly comparable over most of the operating band. The value $angsl=160^{\circ }$ was selected because it provides more consistent broadband impedance matching than $angsl=150^{\circ }$, which exhibits $|S_{11}|<-10.06$ dB over 3.12–21.40 GHz, without causing a meaningful reduction in realized gain.

Figure 2c shows that $W_{f1}$ has a stronger influence on impedance matching than on the lower- and mid-band gain. The selected value $W_{f1}=1.50$ mm provides the most favourable overall impedance response across the targeted operating band while maintaining a realized-gain response comparable to those obtained with the alternative values. Therefore, $W_{f1}=1.50$ mm was selected based primarily on broadband matching rather than on the maximum gain at an individual frequency.

Figure 2d presents the effect of the matching-transformer width, $W_{m}$. The gain curves are nearly identical for the investigated values, indicating that $W_{m}$ has only a limited influence on realized gain. Its optimisation was therefore governed by the impedance response. Although the analytically calculated initial value was $W_{m}=1.51$ mm, the full-wave analysis showed that $W_{m}=2$ mm provides more consistent broadband impedance matching. Accordingly, $W_{m}=2$ mm was selected, while the comparable gain responses confirm that this choice does not introduce a gain penalty.

Similarly, Figure 2e shows that $L_{f1}$ produces only minor changes in realized gain but noticeably modifies the impedance response. Although $L_{f1}=5.76$ mm is close to the analytically calculated value of 5.845 mm, the full-wave analysis showed that $L_{f1}=6.6$ mm provides the most favourable broadband matching while maintaining essentially comparable realized-gain behaviour. The difference between the analytical and optimised values is attributed to the broadband discontinuities, fringing fields, and electromagnetic coupling that are not fully represented by the conventional quarter-wavelength transmission-line model.

**Figure 3:**
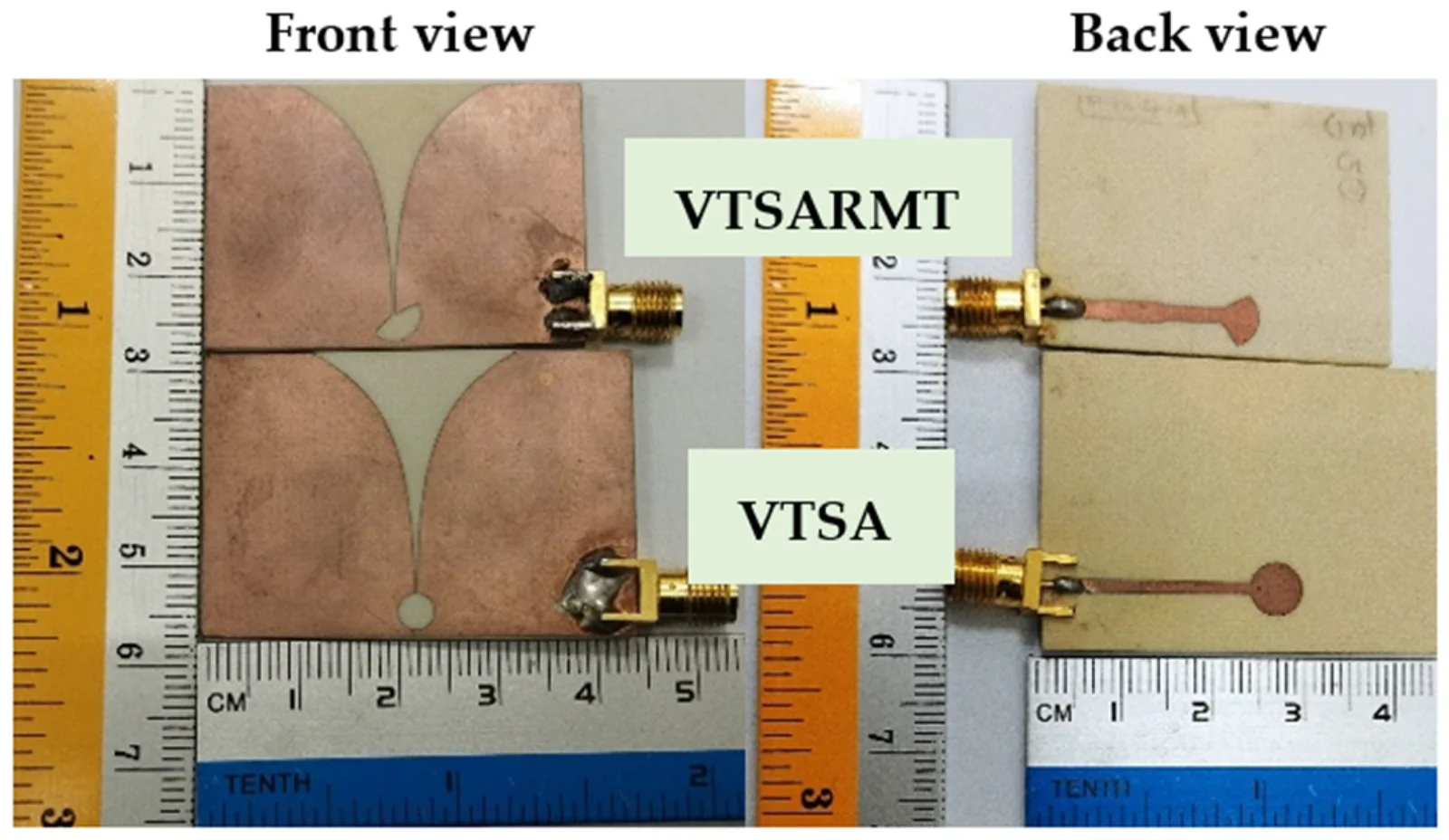
Prototypes of compact UWB VTSARMT as compared to VTSA in [9].

With the final optimized parameters, the VTSARMT achieves a physical footprint area of 1046.76 mm^2^ (36.6×28.6 mm^2^), corresponding to a 16.67% footprint-area reduction relative to the reference VTSA. The simulated operating band extends from 3.13 to 22.05 GHz, compared with 2.95–12.79 GHz for the reference VTSA, corresponding to a 48.00% simulated bandwidth improvement. The maximum simulated realized gain also increases from 6.63 to 7.64 dBi. Table 1 and Figure 3 present the final optimised antenna dimensions and fabricated VTSARMT prototype.

**Figure 4:**
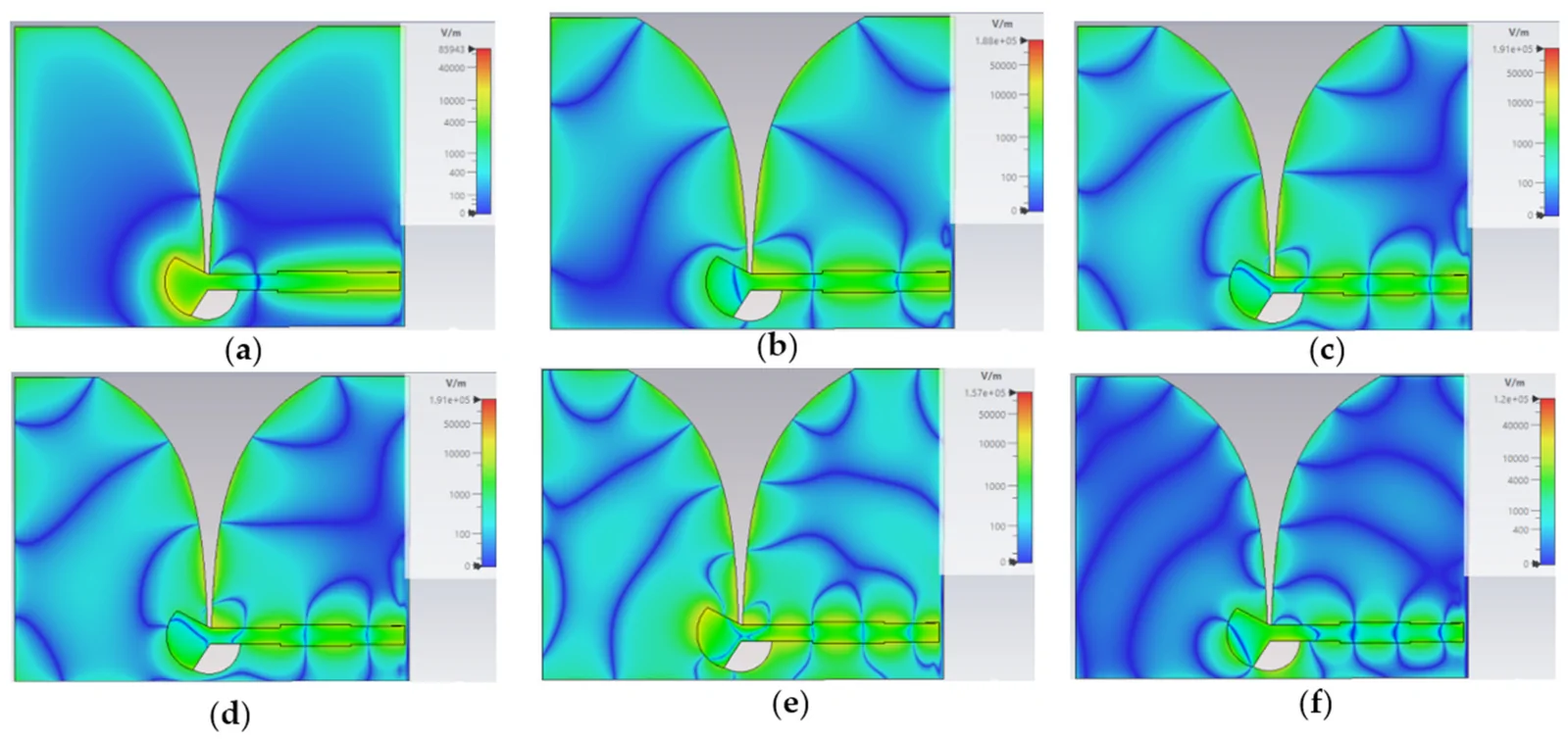
Simulated electric-field magnitude distributions of the standalone VTSARMT at (**a**) 3 GHz, (**b**) 7 GHz, (**c**) 11 GHz, (**d**) 15 GHz, (**e**) 19 GHz, and (**f**) 21 GHz.

To provide physical insight into the proposed feeding structure, Figure 4 presents the simulated electric-field magnitude distributions of the standalone VTSARMT at 3, 7, 11, 15, 19, and 21 GHz, representing the lower, intermediate, and upper regions of the operating band. At 3 GHz, the electric field is broadly distributed around the feeding transition and tapered-slot region. As the frequency increases, additional spatial variations appear along the radial transition, tapered-slot edges, and aperture because the antenna becomes electrically larger. Nevertheless, a continuous field-transfer path remains visible from the 50 Ω input line through the matching transformer and radial-to-radial transition towards the tapered-slot aperture at all selected frequencies. These results demonstrate the broadband field-transfer behaviour of the proposed feeding structure.

**Figure 5:**
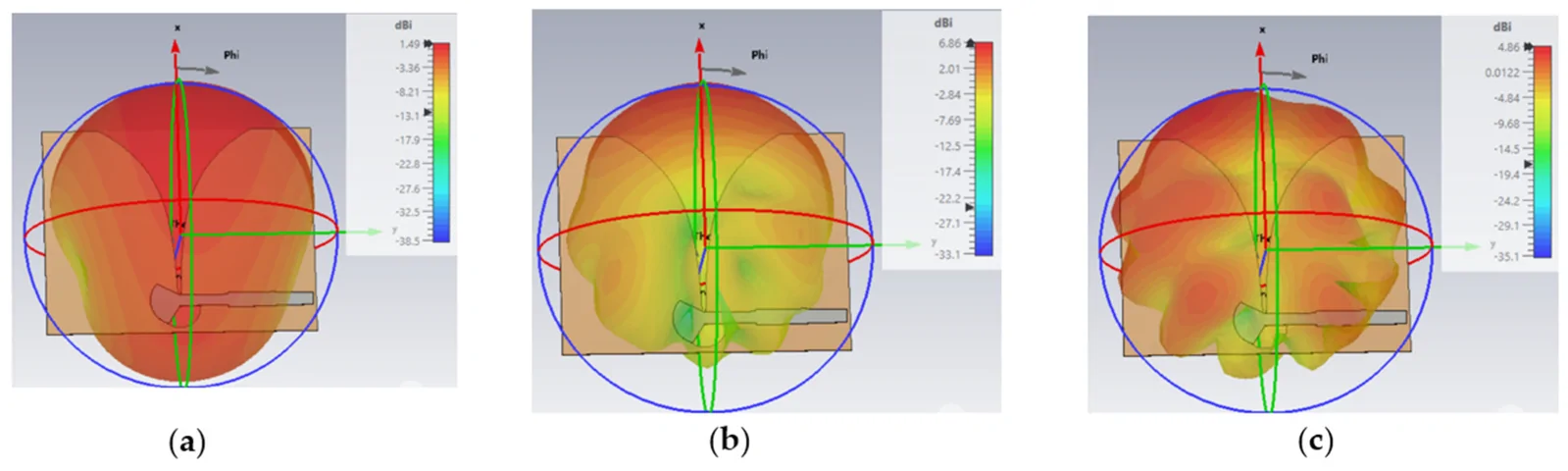
Simulated 3D radiation pattern of the proposed VTSARMT at (**a**) 3 GHz, (**b**) 13 GHz, and (**c**) 19 GHz.

**Figure 6:**
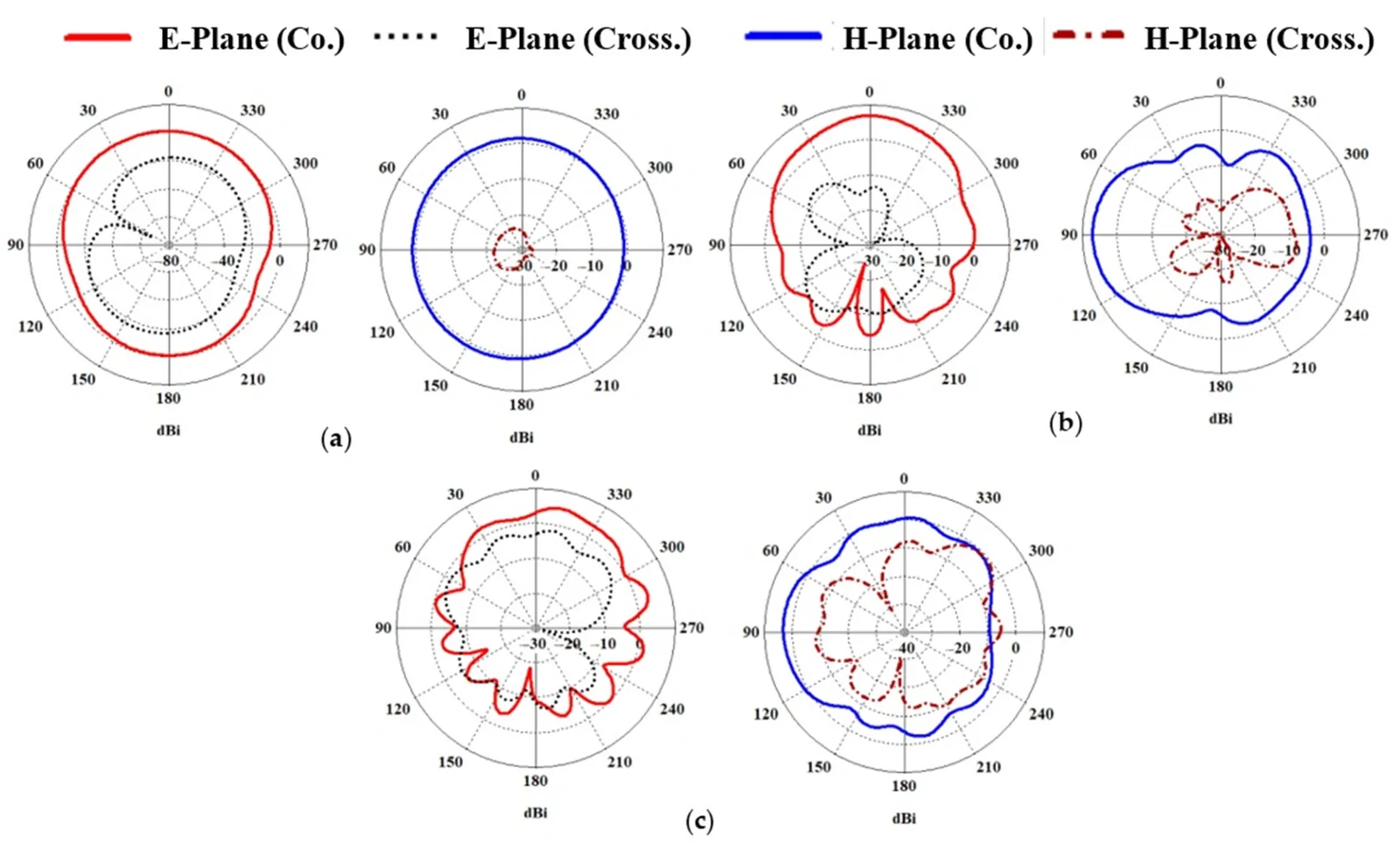
Simulated 2D radiation pattern of the proposed VTSARMT at (**a**) 3 GHz, (**b**) 13 GHz, and (**c**) 21 GHz.

To further evaluate the radiation behaviour of the VTSARMT, Figure 5 and Figure 6 present the simulated 3D realized-gain patterns and 2D co- and cross-polarised E- and H-plane patterns at 3, 13, and 19 GHz. As shown in Figure 5, the antenna exhibits the expected end-fire radiation behaviour, with the dominant radiation directed along the antenna aperture. At 3 GHz, the radiation distribution is comparatively smooth, whereas the patterns become more directive and spatially complex at 13 and 19 GHz, with additional lobes appearing at the higher frequencies. Figure 6 further examines the corresponding co- and cross-polarised components. The co-polarised component is dominant at 3 GHz, while increased angular variation and cross-polarisation are observed at 13 and 19 GHz. This behaviour is attributed to the electrically larger aperture and more complex field and edge-current distributions at higher frequencies. The increased cross-polarization may reduce polarization purity and received power in polarization-matched links; however, it does not directly alter the reported impedance bandwidth [52]. Future work will investigate transition symmetry, edge corrugations, and balanced feeding to suppress the upper-band cross-polarized component.

## 3. On-Vehicle Integration and Stationary Directional Link Assessment

The proposed VTSARMT was numerically evaluated in CST Microwave Studio to investigate the effects of rooftop integration and nearby vehicle bodies on its electromagnetic response. All results in this section are simulated, while the experimental validation in Section 4 concerns the standalone antenna. The analysis considers stationary, orientation-controlled configurations and is intended as a comparative electromagnetic assessment rather than a complete dynamic vehicular-channel validation. Because the VTSARMT exhibits end-fire radiation, the antennas were oriented towards the intended receiving vehicles. The investigated configurations therefore represent selected directional links rather than continuous omnidirectional coverage. Vehicle motion, arbitrary orientations, beam steering, dynamic blockage, and time-varying multipath propagation are outside the scope of this study.

The simulated bandwidth of 3.13–22.05 GHz represents the broadband antenna response and does not imply that the entire range is allocated for automotive communication. The proposed VTSARMT is not intended as an alternative to a 77–81 GHz automotive radar antenna. Any practical implementation must comply with the frequency allocations and emission limits applicable to the intended service. Accordingly, this section first examines the effects of vehicle integration and the two- and three-vehicle configurations, followed by the transmission-coefficient and received-power analysis of the selected stationary directional links.

### 3.1. Vehicle Integration, Configurations, and Radiation Coverage

**Figure 7:**
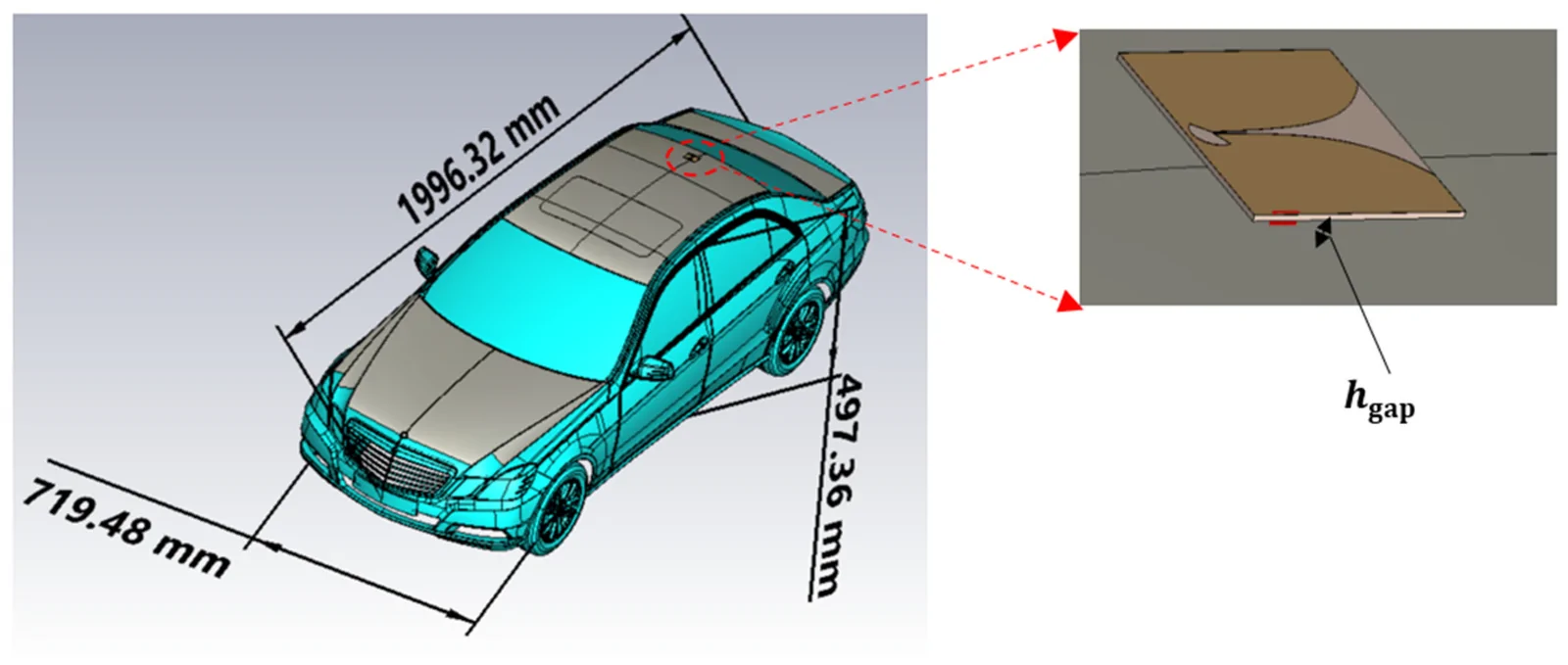
CST passenger-vehicle model showing the vehicle dimensions, rooftop antenna position, and the 4 mm antenna-to-roof air gap in the inset.

A detailed passenger-vehicle model was imported into CST Microwave Studio and uniformly reduced by 50% to obtain a computationally manageable geometry while retaining the complete VTSARMT structure. The final simulated vehicle dimensions were $1996.42\times 719.48\times 497.36\;{mm}^{3}\left(L_{v}(Vehicle\;length)\times W_{v}(Vehicle\;width)\times H_{v}(Vehicle\;height)\right)$ as shown in Figure 7. Because only the vehicle body was reduced while the antenna dimensions and operating frequencies remained unchanged, the model is used for comparative assessment rather than as an electromagnetically scaled representation of a full-size production vehicle.

The principal metallic parts of the vehicle were modelled as perfect electric conductors, while the windscreen and windows were represented using a simplified dielectric-glass model with $\varepsilon_{r}=6.5$ and tanδ=0.02. The VTSARMT retained its original conductor and Rogers RO4003C substrate properties. The antenna was positioned above the rear-central roof region. A uniform air gap of $h_{gap}=4$ mm was maintained between the lower substrate surface and the metallic roof, as illustrated in the inset of Figure 7. The antenna location, height, orientation, vehicle materials, boundaries, solver settings, and mesh criteria were maintained consistently throughout the comparisons.

**Figure 8:**
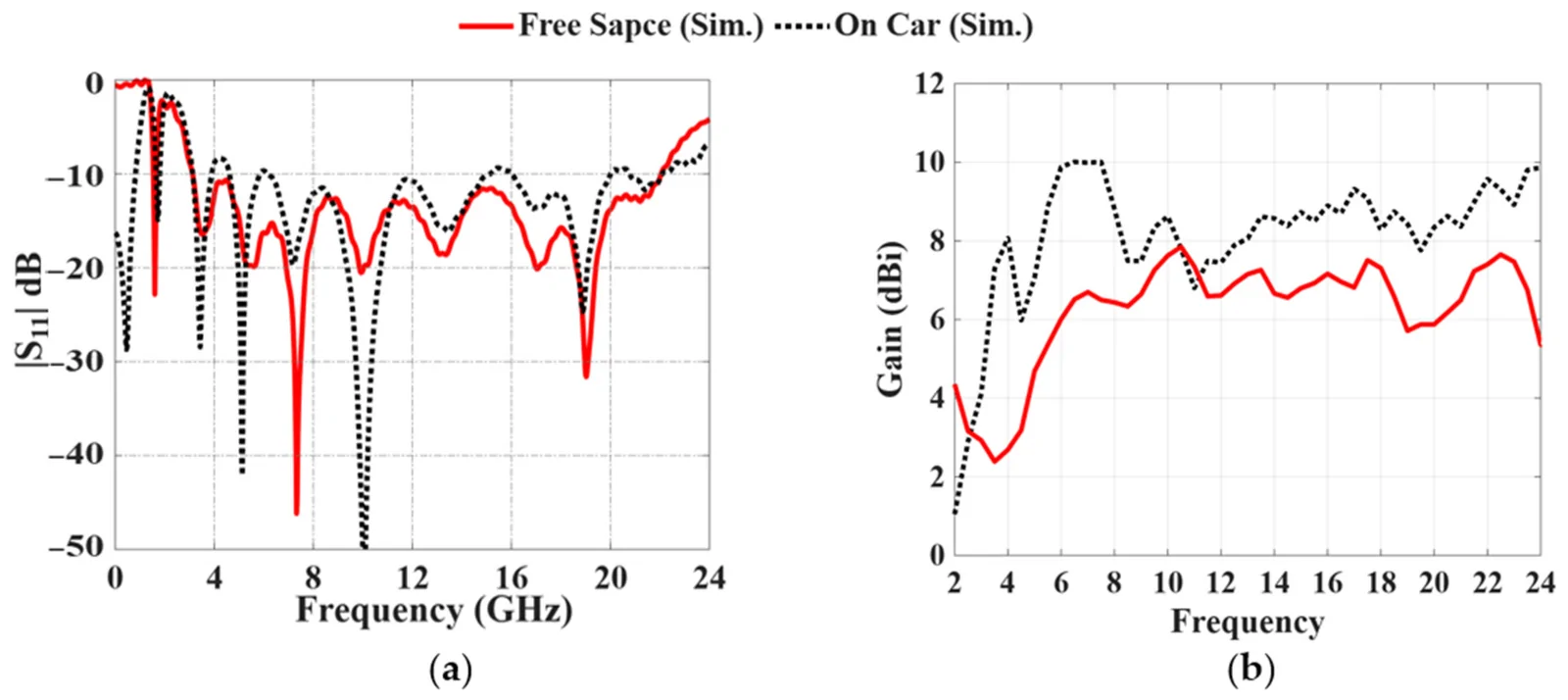
Comparison of the proposed VTSARMT in free space and after rooftop integration: (**a**) simulated reflection coefficient and (**b**) simulated realized gain.

The effect of rooftop integration was evaluated by comparing the standalone and rooftop-mounted antennas. Figure 8 compares the simulated reflection coefficient and realized gain of the standalone and rooftop-mounted VTSARMT. Rooftop integration produces frequency-dependent shifts in the resonances and changes in the matching level because the conductive vehicle body modifies the antenna near field and input impedance. The overall broadband response remains evident, although the exact on-vehicle operating band differs from the free-space result. The rooftop-mounted antenna also exhibits a generally higher realized gain over much of the investigated frequency range. This behaviour is associated with induced vehicle-body currents and field reflection, which redistribute the radiation and increase directivity in selected directions. The result should therefore be interpreted as a change in the integrated antenna–vehicle radiation response rather than a uniform gain improvement in all directions.

**Figure 9:**
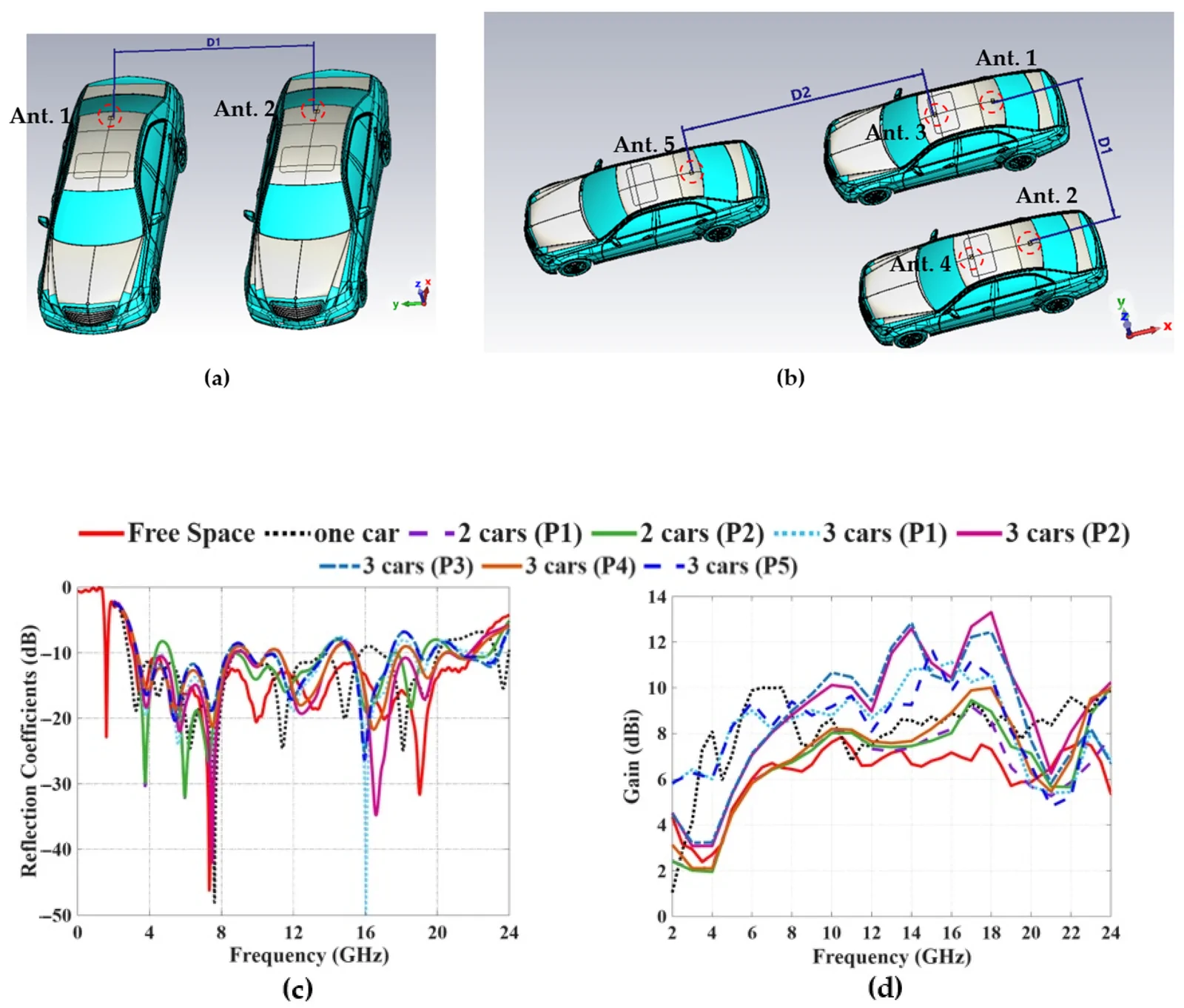
Vehicle configurations and performance comparison: (**a**) two-vehicle; (**b**) three-vehicle; (**c**) simulated $S_{11}$; and (**d**) realized gain for free-space and on-vehicle cases.

Following the rooftop-integration assessment, two stationary vehicle configurations were investigated, as shown in Figure 9. The first consists of two vehicles positioned side by side and represents a lateral directional link. The second consists of three vehicles and combines lateral and longitudinal directional paths within the same model. Owing to the end-fire radiation characteristic and the longitudinal separation between the vehicles, additional antennas were positioned near the front and rear roof regions in the three-vehicle configuration. This arrangement provides selected directional paths for Ant. 3, Ant. 4, and Ant. 5 and is used only for the present stationary assessment. It is not intended to represent a complete omnidirectional or MIMO vehicular antenna system. To quantify the influence of vehicle integration and the additional surrounding vehicles, Figure 9c,d compare the $S_{11}$ and realized-gain responses of the proposed antenna in free space and in the single-, two-, and three-vehicle configurations. As shown in Figure 9c, all configurations generally retain broadband impedance matching, although the vehicle environment produces clear frequency-dependent shifts in the resonance locations and matching levels. For example, several additional or shifted resonance minima appear around 4–8 GHz and 16–19 GHz, with some vehicle-mounted cases reaching approximately −30 to below −40 dB, compared with the shallower free-space response at the corresponding frequencies. This confirms that the surrounding conductive vehicle bodies significantly modify the antenna input response rather than simply producing a uniform matching improvement. The realized-gain comparison in Figure 9d shows a clearer quantitative vehicle-body effect. The free-space antenna exhibits a maximum realized gain of approximately 7.5 dBi, whereas the single-vehicle case reaches approximately 10 dBi. The two-vehicle configurations reach approximately 9–9.5 dBi, while the three-vehicle responses show stronger position-dependent variations, with peak values of approximately 12.5–13.5 dBi for some antenna locations around 14–18 GHz. Conversely, other antenna positions remain closer to approximately 9–11 dBi. The additional vehicles therefore do not produce a uniform gain enhancement; instead, the surrounding conductive bodies redistribute the radiation through reflection and scattering, resulting in frequency- and position-dependent gain changes. The corresponding inter-vehicle transmission coefficients and received-power responses are evaluated separately in the following link analysis.

**Figure 10:**
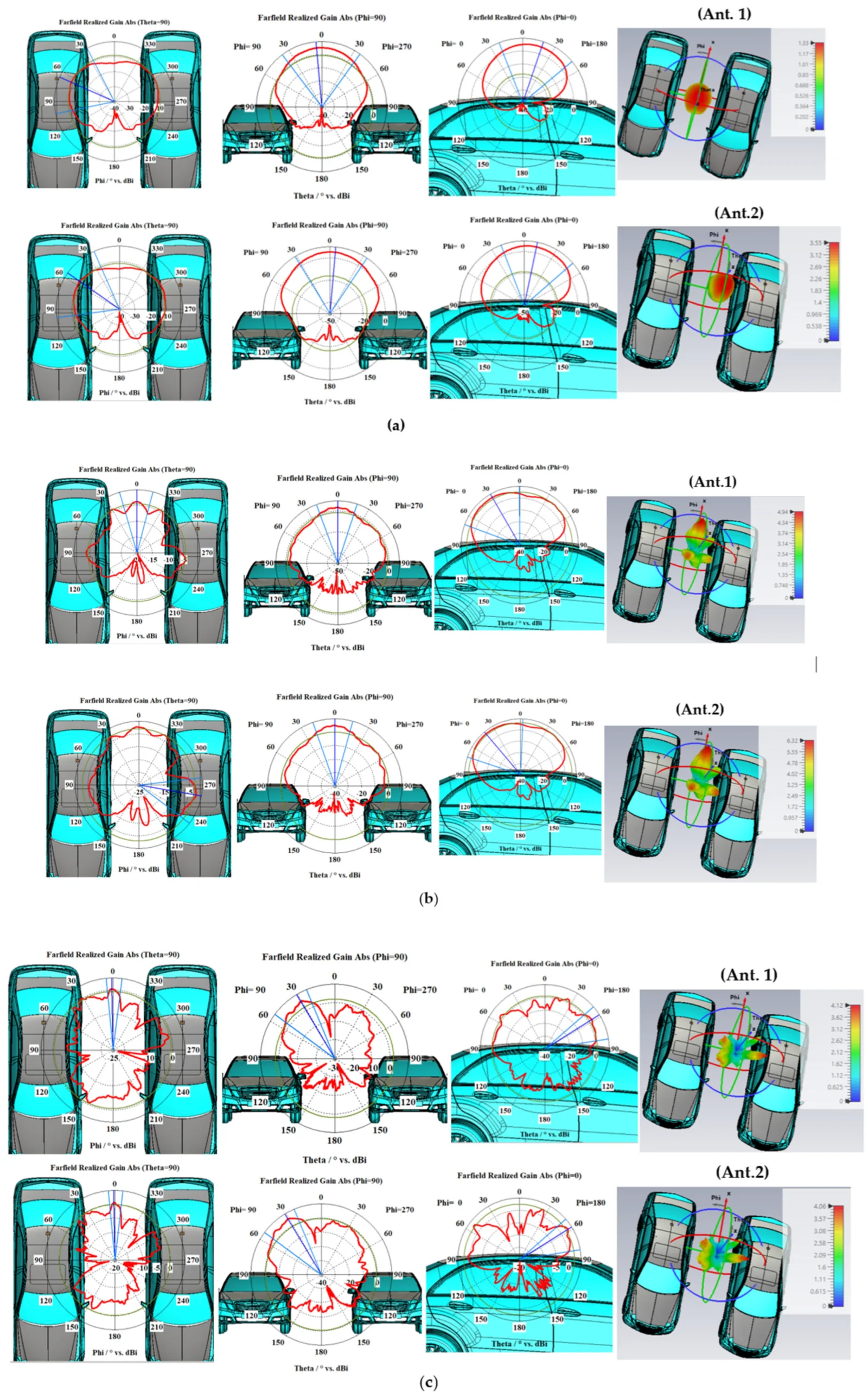

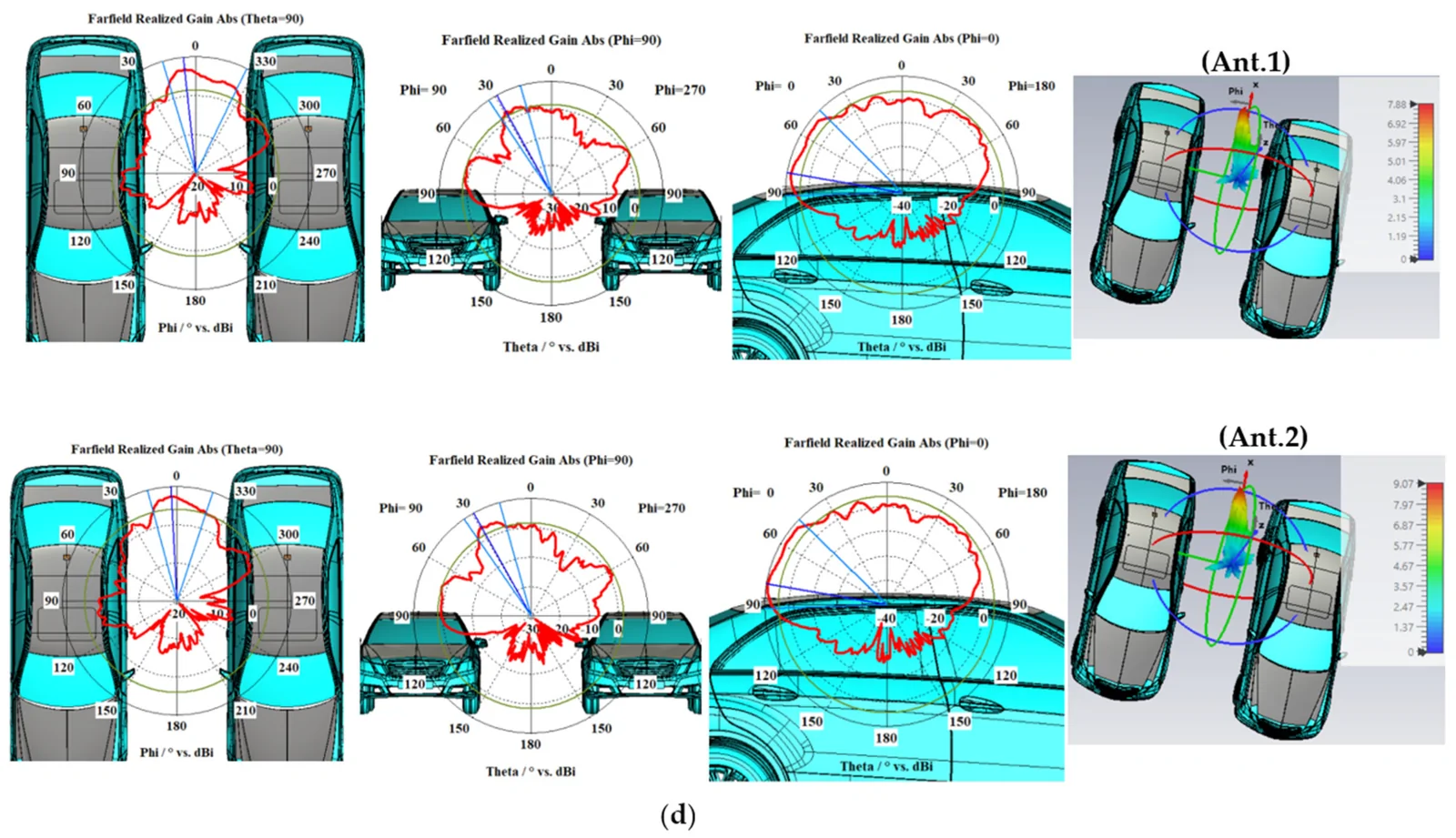
Simulated 3D and 2D radiation patterns of the proposed rooftop-mounted antenna for the two-vehicle side-by-side configuration at: (**a**) 5 GHz, (**b**) 9 GHz, (**c**) 15 GHz, and (**d**) 21 GHz, with D1= 1.2 m.

**Figure 11:**
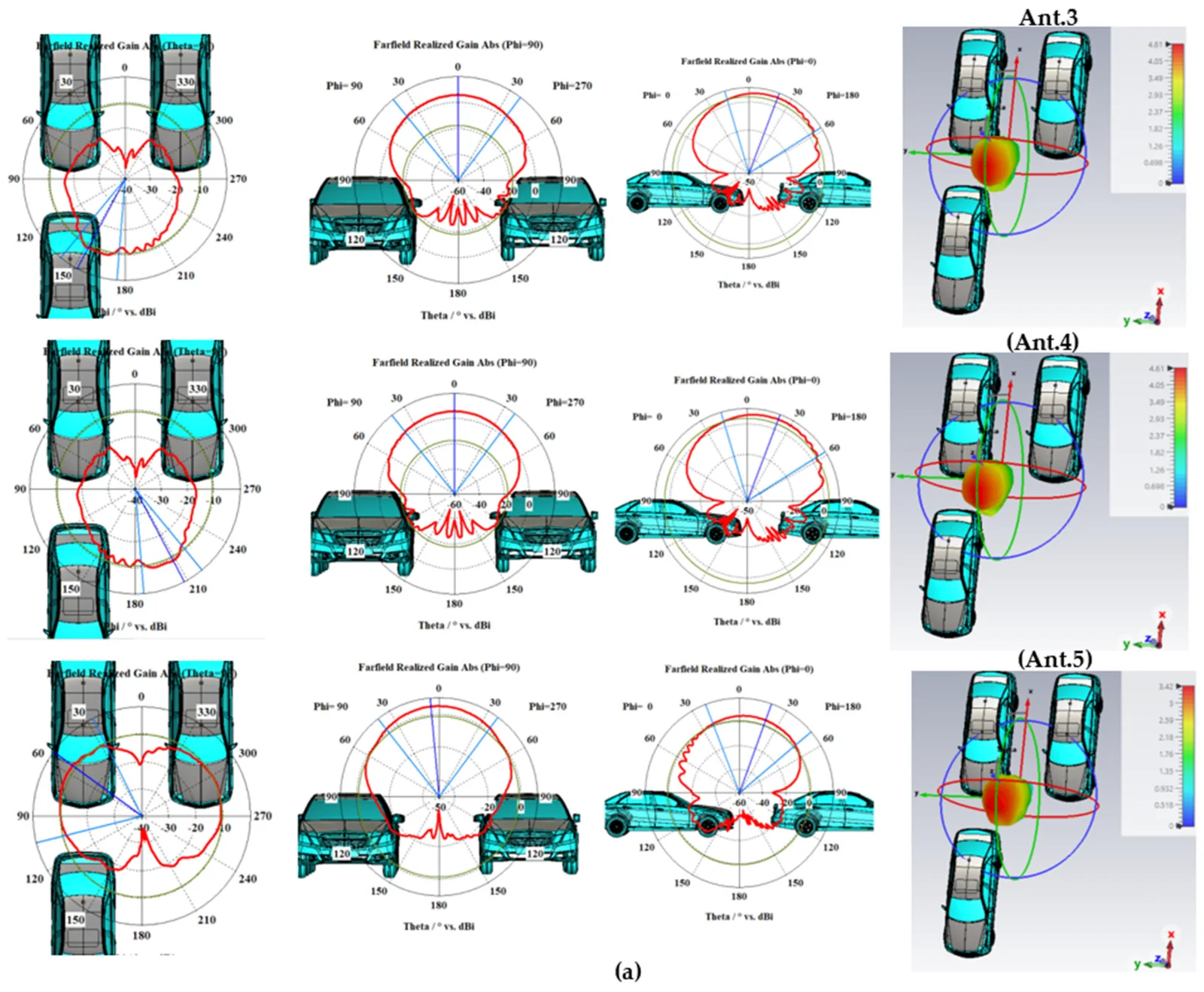

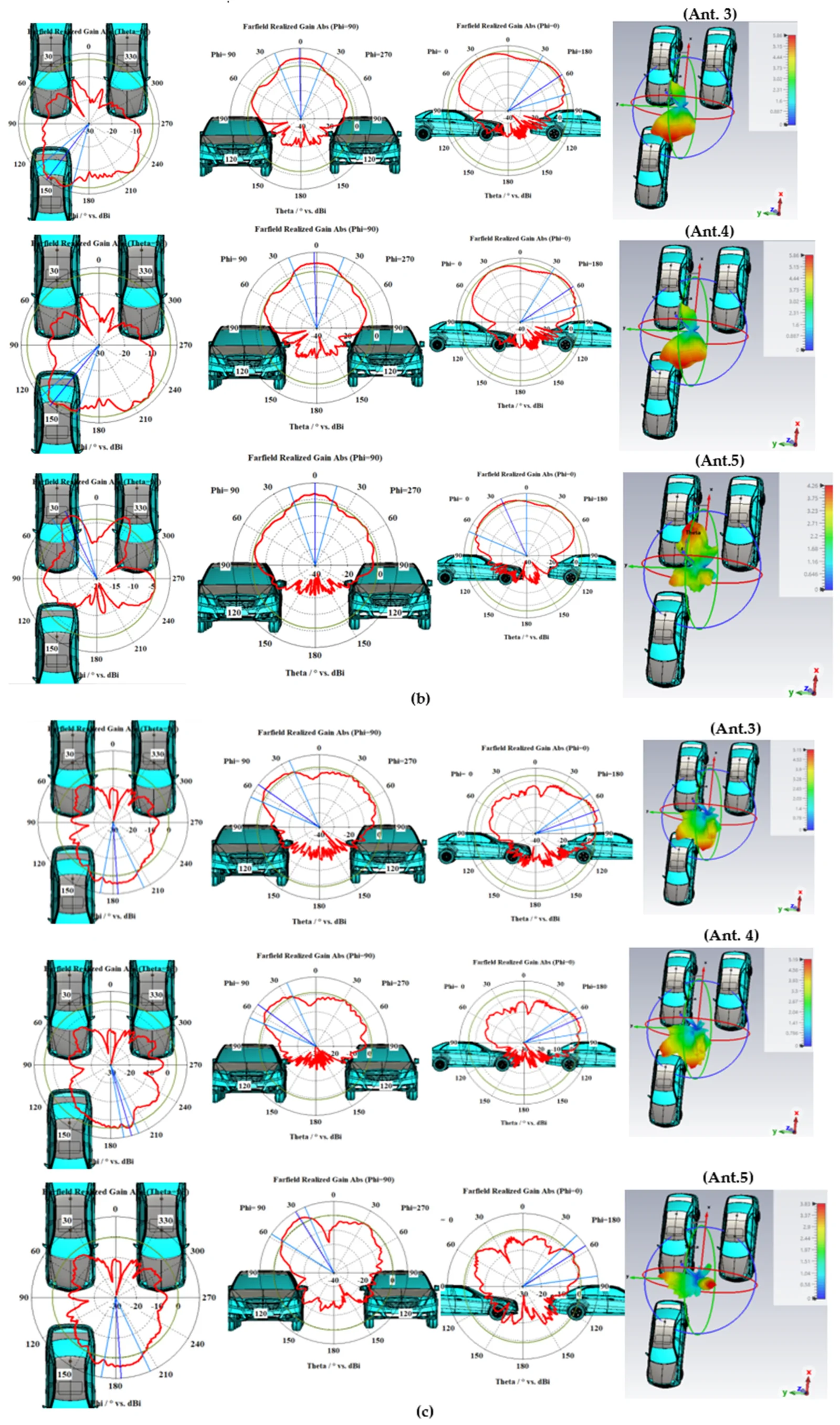

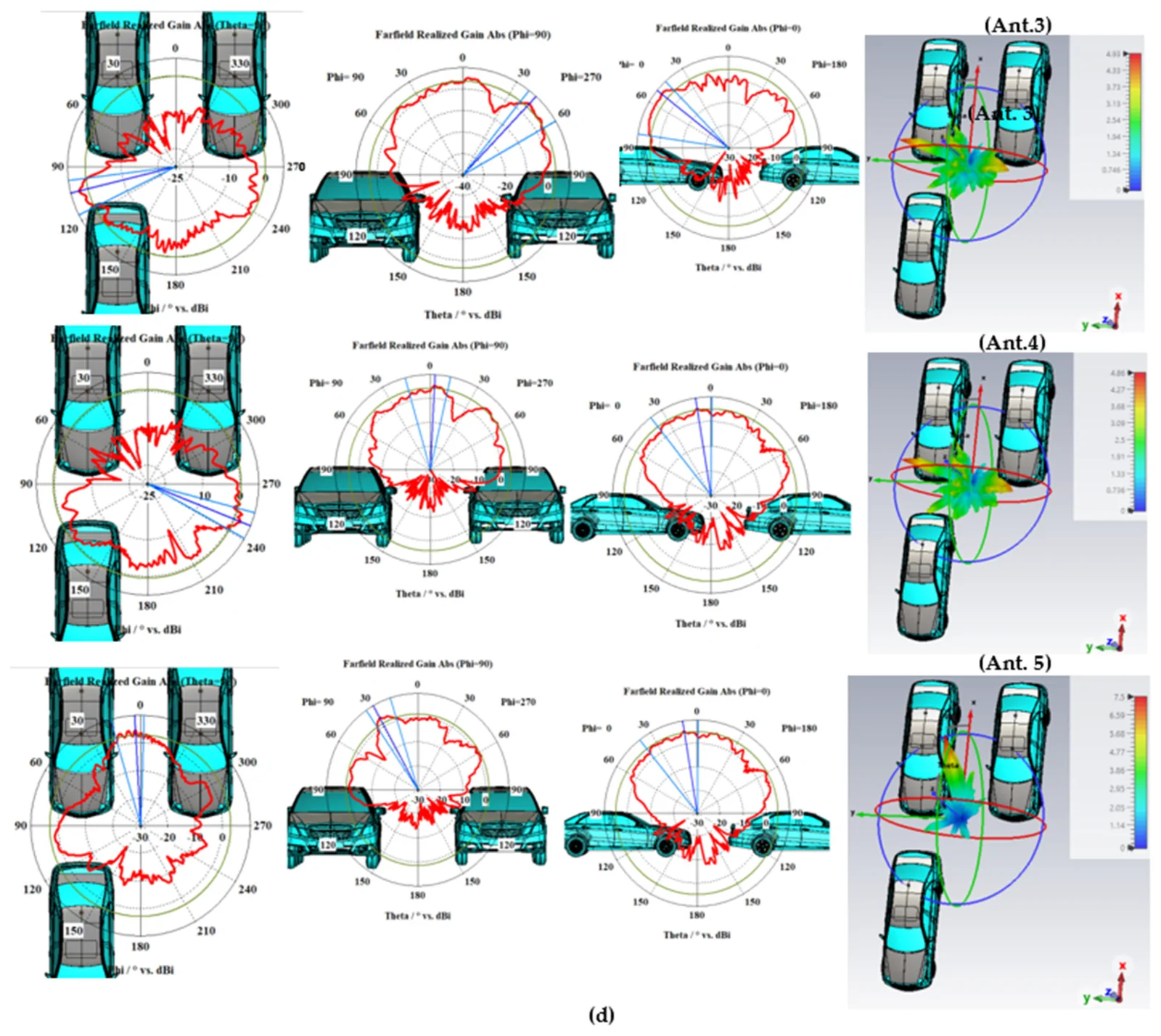
Simulated 3D and 2D radiation patterns of the proposed rooftop-mounted antenna for the three-vehicle configuration at: (**a**) 5 GHz, (**b**) 9 GHz, (**c**) 15 GHz, and (**d**) 21 GHz, with D1 = D2 = 1.2 m.

The radiation analysis was performed for the fixed configuration shown in Figure 9, with antenna-to-antenna separations of $D_{1}=D_{2}=1.2$ m. The influence of varying the separation distance is examined separately through the transmission coefficients in the following subsection. The on-vehicle radiation characteristics were evaluated at 5, 9, 15, and 21 GHz, representing the lower, intermediate, and upper regions of the rooftop-mounted operating band. Figure 10 and Figure 11 present the two-vehicle and three-vehicle results, respectively. In the two-vehicle configuration, the patterns assess the directional coverage between Ant. 1 and Ant. 2. In the three-vehicle configuration, the front- and rear-roof antenna placements provide complementary directional regions for the Ant. 5–Ant. 3 and Ant. 5–Ant. 4 paths.

At the selected frequencies, the vehicle bodies modify the radiation patterns through reflection, blockage, and scattering. The radiation distributions become more spatially complex at 15 and 21 GHz, where the antenna and vehicle geometries are electrically larger. Nevertheless, radiation towards the intended receiving regions is maintained for the investigated stationary configurations. These patterns identify the possible directional paths, while the following transmission analysis quantitatively evaluates the corresponding antenna-to-antenna links.

### 3.2. Transmission and Link Analysis

**Figure 12:**
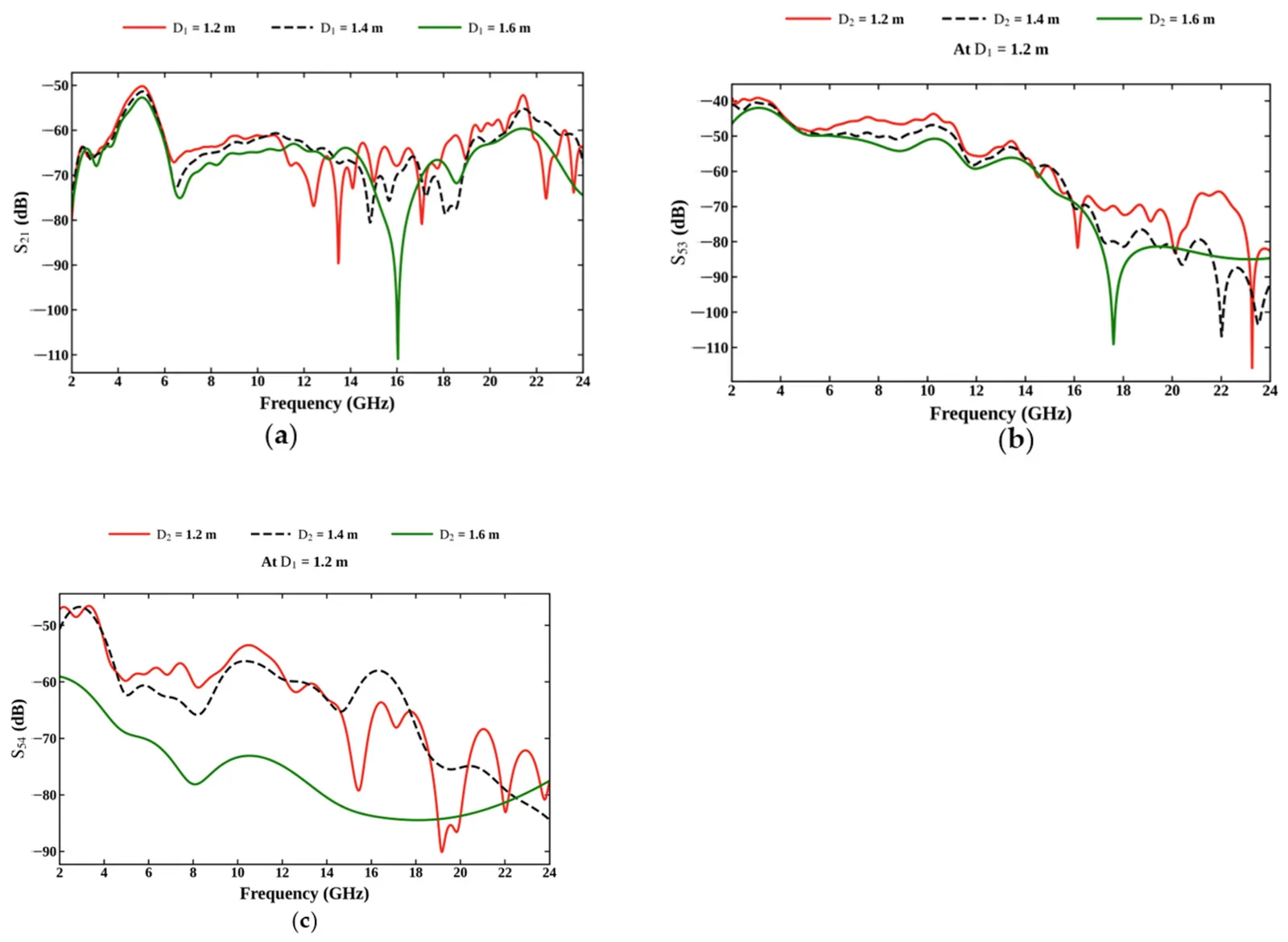
Simulated transmission coefficients for the complete three-vehicle configuration: (**a**) $S_{21}$ for the lateral Ant. 1–Ant. 2 path, (**b**) $S_{53}$ for the longitudinal Ant. 3–Ant. 5 path, and (**c**) $S_{54}$ for the diagonal Ant. 4–Ant. 5 path.

The radiation patterns were evaluated using the fixed initial geometry at $D_{1}=D_{2}=\;1.2$ m. The complete three-vehicle configuration was then used for the transmission analysis to avoid repeating the equivalent two-vehicle case and to evaluate the selected lateral, longitudinal, and diagonal paths within a single simulation environment. In the three-vehicle configuration, $D_{1}$ denotes the antenna-to-antenna separation between Ant. 1 and Ant. 2, while $D_{2}$ denotes the separation between Ant. 3 and Ant. 5. The investigated values were $D_{1}=D_{2}=1.2,\;1.4,\;$ and 1.6 m. For the $S_{21}$ analysis, $D_{1}$ was varied. For the $S_{53}$ and $S_{54}$ analyses, $D_{2}$ was varied while $D_{1}$ was fixed at 1.2 m. Thus, $S_{21}$ represents the lateral Ant. 1–Ant. 2 path, $S_{53}$ represents the longitudinal Ant. 3–Ant. 5 path, and $S_{54}$ represents the diagonal Ant. 4–Ant. 5 path under the corresponding $D_{2}$ configurations. A less negative $S_{ij}$ value indicates stronger port-to-port transmission. Figure 12 shows that the transmission coefficients generally become more negative as the corresponding separation increases, indicating reduced power transfer between the rooftop-mounted antennas. Frequency-dependent fluctuations and deep minima are also observed because of propagation loss, directional-pattern variation, beam overlap, and reflection, blockage, diffraction, and scattering from the vehicle bodies. At higher frequencies, increased free-space loss and the electrically larger vehicle geometry contribute to weaker transmission. The results are interpreted as comparative stationary, orientation-controlled transmission responses rather than validation of a complete dynamic V2V channel.

For matched and consistently power-normalised CST ports, the transmission-derived attenuation was calculated as

(2) $$\begin{array}{cccc} & PL_{CST}\left(dB\right)=-S_{ij}\left(dB\right), & & \end{array}$$

where $S_{ij}$ is the simulated transmission coefficient from transmitting port j to receiving port i. The corresponding received power was calculated as

(3) $$\begin{array}{cccc} & P_{r,\;CST}\left(dBm\right)=P_{t}\left(dBm\right)-PL_{CST}\left(dB\right), & & \end{array}$$

or equivalently,

(4) $$\begin{array}{cccc} & P_{r,\;CST}\left(dBm\right)=P_{t}\left(dBm\right)+S_{ij}\left(dB\right). & & \end{array}$$

The simulated $S_{ij}$ response incorporates the antenna matching, realized radiation behaviour, antenna orientation, rooftop mounting, vehicle-body interaction, separation distance, and surrounding geometry represented in CST. Antenna gains were therefore not added separately to the CST-based received-power calculation, as this would double-count their contribution.

**Figure 13:**
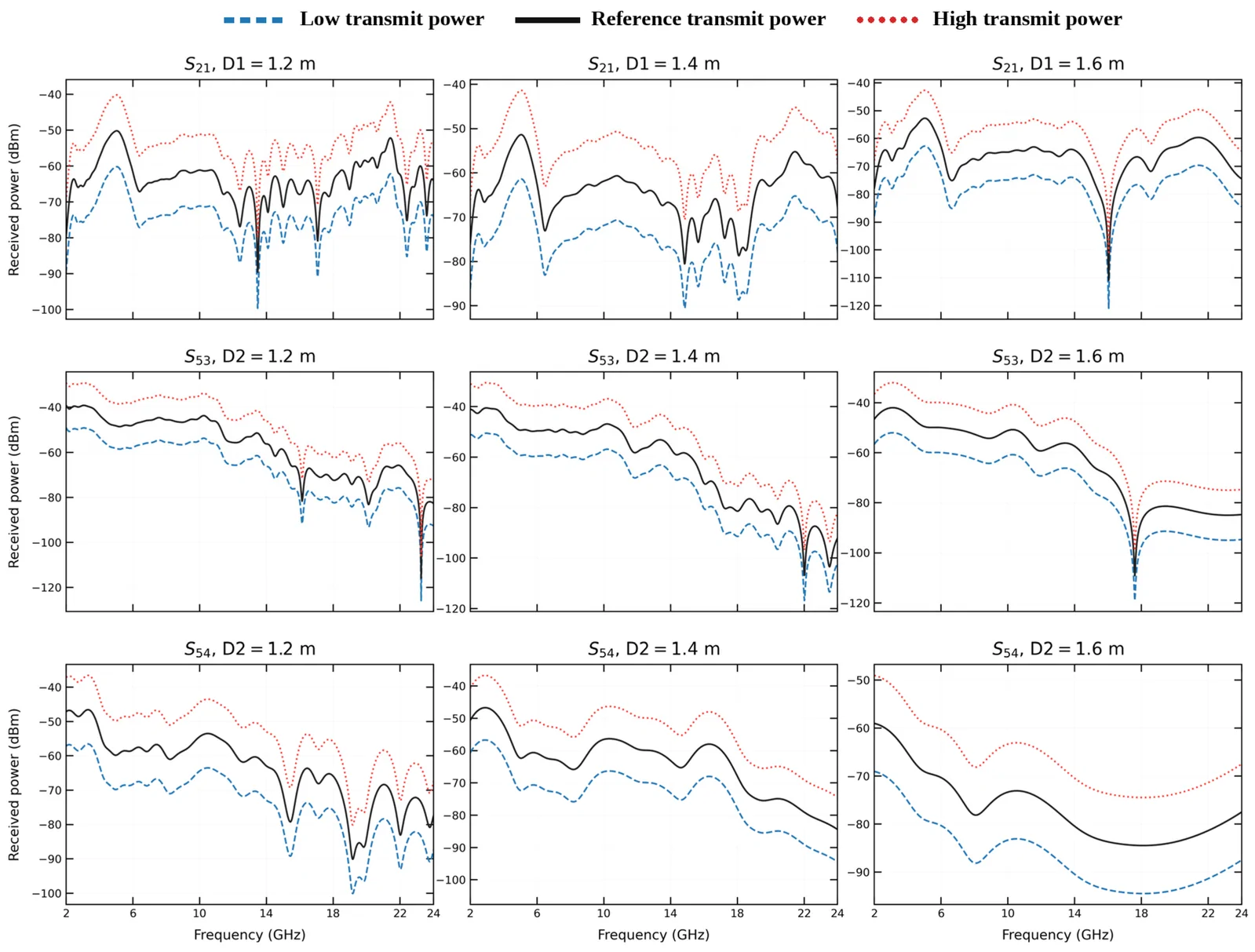
CST-based received power for the investigated stationary directional links at antenna-to-antenna separations of 1.2, 1.4, and 1.6 m under low, reference, and high transmit-power assumptions of −10, 0, and +10 dBm, respectively.

Figure 13 presents the CST-based received power for the $S_{21}$, $S_{53}$, and $S_{54}$ links at the investigated antenna separations. The low, reference, and high transmit-power cases correspond to $P_{t}=-10$, 0, and +10 dBm, respectively. Since only $P_{t}$ changes, the three responses have identical frequency-dependent shapes and are separated by 10 dB.

The received power generally decreases with increasing separation and towards the upper-frequency region. However, the curves do not vary monotonically at every frequency. The local fluctuations and deep minima result from the combined effects of frequency-dependent radiation-pattern overlap, propagation loss, destructive interference, and vehicle-body reflection, blockage, diffraction, and scattering. The particularly deep minima indicate frequency-selective nulls rather than distance-dependent attenuation alone.

Among the investigated paths, $S_{21}$ exhibits pronounced frequency-dependent variations across the band. The $S_{53}$ response decreases more noticeably towards the upper frequencies, while the $S_{54}$ response changes with the corresponding $D_{2}$ geometry and becomes weaker for the largest separation. These results represent comparative received powers at the simulated ports for stationary, orientation-controlled configurations. They do not include cable losses, receiver sensitivity, receiver noise figure, modulation requirements, vehicle motion, or time-varying multipath propagation and therefore do not establish a practical communication range.

### 3.3. Limitations and Future Work

The on-vehicle results in this work are limited to stationary, orientation-controlled CST configurations using a uniformly reduced vehicle model. The analysis does not include full-scale vehicle-mounted measurements, vehicle motion, changing antenna alignment, road and infrastructure reflections, surrounding traffic, dynamic blockage, receiver sensitivity, or time-varying multipath propagation. The results should therefore be interpreted as a comparative assessment of rooftop integration and stationary directional links rather than validation of a complete practical V2V channel. Future work will investigate full-scale vehicle measurements and dynamic propagation conditions. Wider and adaptive angular coverage will also be explored using sector-oriented Vivaldi arrays, diversity/MIMO configurations, and beam-steerable architectures. Vivaldi-based beam-steerable arrays have previously demonstrated controllable directional coverage for automotive applications, while reconfigurable leaky-wave antennas provide another potential low-profile beam-steering approach [52]. Alternative mounting positions, including vehicle-window integration, will also be investigated with consideration of glass properties, mutual coupling, isolation, correlation, and combined radiation patterns.

## 4. Results and Discussion

This section presents the experimental validation of the standalone fabricated VTSARMT and provides a direct comparison between the simulated and measured antenna performance. The comparison includes $S_{11}$, realized gain, radiation patterns, phase, total radiation efficiency, and group delay, with the reference VTSA in [9] included where applicable.

**Figure 14:**
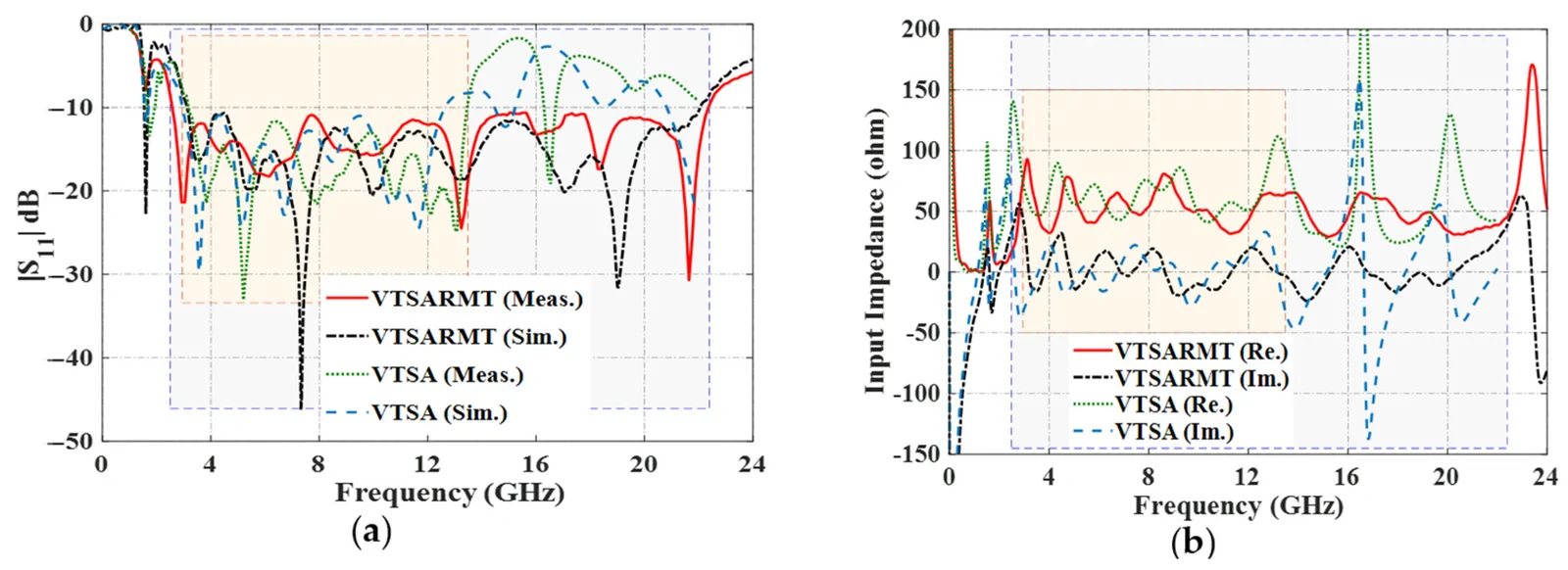
(**a**) Simulated and measured S_11_ and (**b**) simulated input impedance of compact UWB VTSARMT as compared to VTSA in [9].

**Figure 15:**
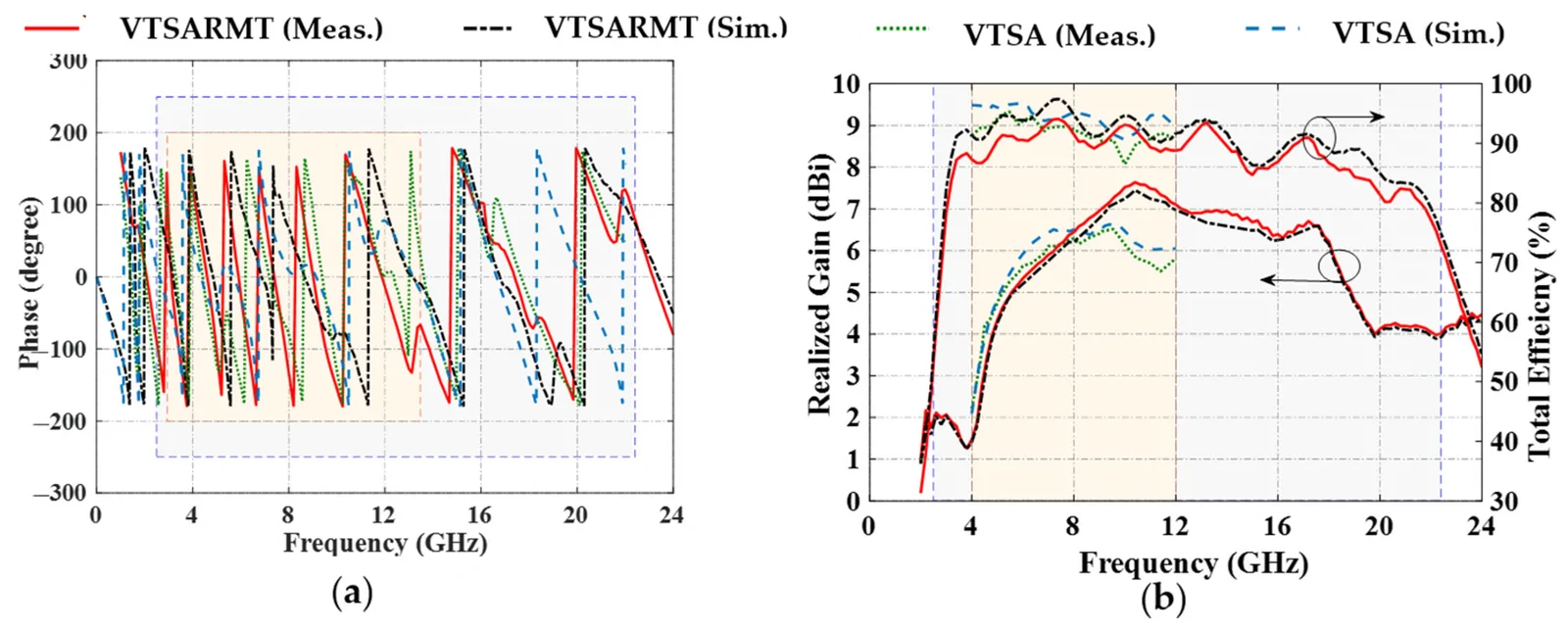
Simulated and measured (**a**) phase and (**b**) gain with total radiation efficiency of compact UWB VTSARMT as compared to VTSA in [9].

The measured data of the proposed VTSARMT, compared with the VTSA in [9], are illustrated in Figure 14 and Figure 15. Figure 14a and Figure 15b show that the proposed compact UWB VTSARMT provides good, measured impedance matching of $|S_{11}|\;<-10.61$ dB over 2.58–22.34 GHz and a maximum realized gain (MRG) of 7.45 dBi. Compared with the measured VTSA in [9], the proposed VTSARMT achieves a 47.67% measured bandwidth improvement and a 16.67% footprint-area reduction, while the maximum measured realized gain increases from 6.51 to 7.45 dBi.

In Figure 14b, the simulated real and imaginary parts of the input impedance oscillate around 50 Ω and 0 Ω, respectively, which explains the good impedance-matching behaviour observed in Figure 14a. Figure 15a shows a slight phase-shift difference between the VTSA and VTSARMT. The measured total radiation efficiency of the VTSARMT, shown in Figure 15b, ranges from 47.7% to 94.08%, while the simulated efficiency ranges from 49.09% to 97.43%.

**Figure 16:**
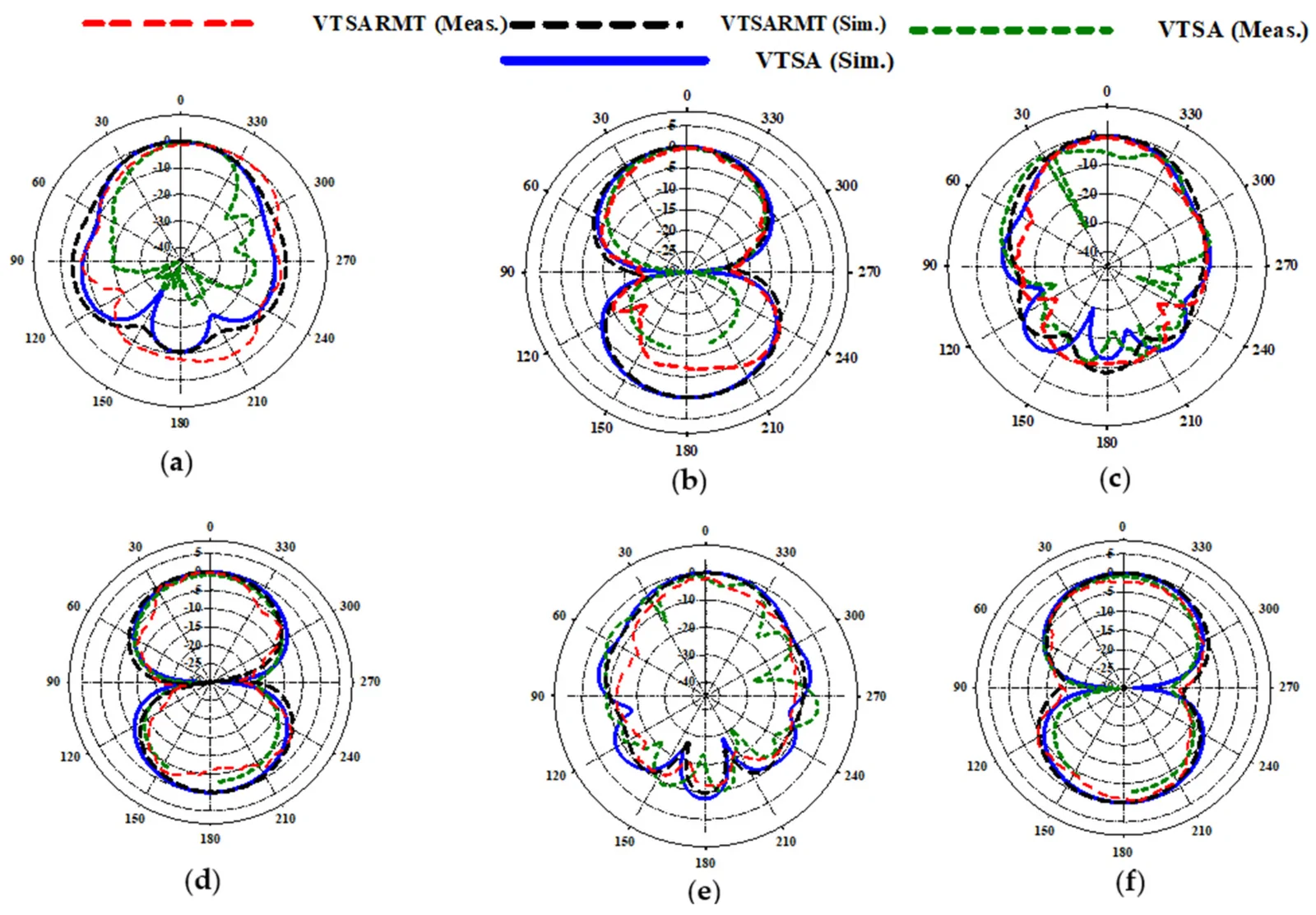
Simulated and measured radiation patterns of compact UWB VTSARMT as compared to VTSA in [9] at 5.11 GHz (**a**) E and (**b**) H, 8.2 GHz (**c**) E and (**d**) H, and 10 GHz (**e**) E and (**f**) H.

**Figure 17:**
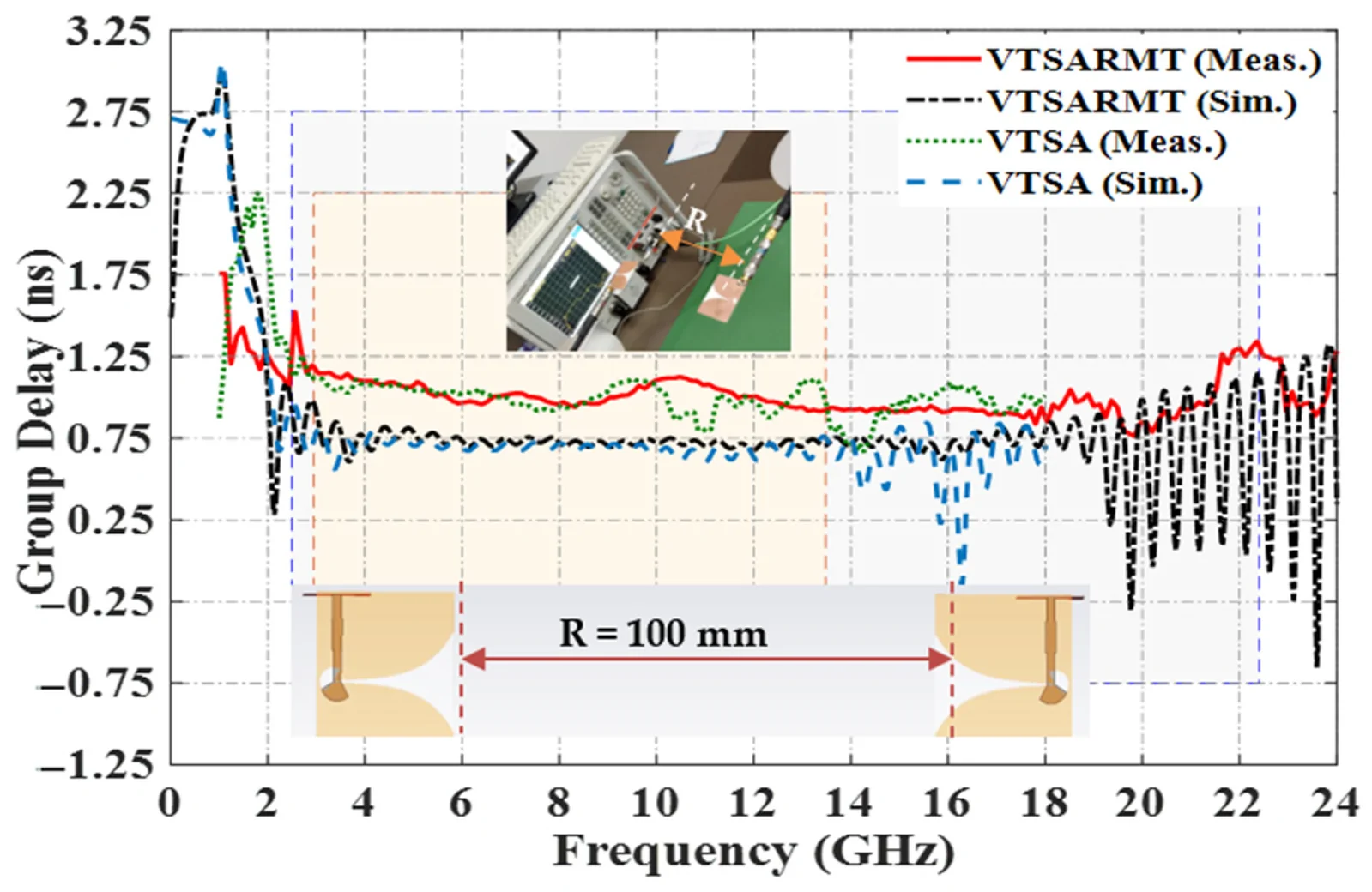
Simulated and measured group delay of compact UWB VTSARMT as compared to VTSA in [9].

Figure 16 compares the simulated and measured principal-plane radiation patterns of the proposed VTSARMT with those of the reference VTSA at 5.11, 8.2, and 10 GHz. These frequencies were selected because they lie within the common measured operating range of both antennas, thereby enabling a consistent comparison of their radiation characteristics. The proposed VTSARMT maintains the characteristic directional behaviour of a Vivaldi antenna, with predominantly end-fire radiation in the E-plane and dipole-like behaviour in the H-plane. The measured patterns generally follow the simulated trends, although some variations in the sidelobe levels and pattern symmetry are observed. These differences may be associated with fabrication tolerances, connector and cable effects, and measurement-environment uncertainties, which are not fully represented in the simulation. Finally, Figure 17 shows that the proposed VTSARMT provides an approximately stable measured group delay of 1 ns, compared with a simulated value of approximately 0.7 ns, across most of the operating bandwidth. A higher level of dispersion is observed above 20 GHz, which can be attributed to increased losses at higher frequencies. Figure 14, Figure 15, Figure 16 and Figure 17 confirm the effectiveness of using the radial-to-radial transition together with the matching transformer to reduce the size of the VTSA while enhancing both bandwidth and gain without introducing additional complex matching circuits or parasitic elements. These results experimentally validate the standalone broadband antenna performance, while the CST-based analysis in Section 3 provides a preliminary assessment of its rooftop integration and stationary directional-link behaviour.

Table 2 compares the proposed VTSARMT with recent compact and performance-enhanced Vivaldi antennas reported in the literature. To provide a consistent comparison among antennas operating at different frequencies and on different substrates, fractional bandwidth (FBW) and antenna dimensions normalised to the guided wavelength at the lower operating frequency of each design are reported. The FBW and guided wavelength are calculated as

(5) $$\begin{array}{cccc} & FBW\left(\%\right)=\frac{2\left(f_{H}-f_{L}\right)}{f_{H}+f_{L}}\times 100, & & \end{array}$$

and

(6) $$\begin{array}{cccc} & \lambda_{g}=\frac{c}{f_{L}\sqrt{\varepsilon_{r}}}, & & \end{array}$$

where $f_{H}$ and $f_{L}$ are the upper and lower operating frequencies, respectively, c is the speed of light in free space, and $\varepsilon_{r}$ is the substrate relative permittivity.

The proposed VTSARMT achieves a measured operating band of 2.58–22.34 GHz, corresponding to an FBW of 158.59%, with normalised dimensions of $0.593\lambda_{g}\times 0.464\lambda_{g}\times 0.013\lambda_{g}$ and a maximum measured realised gain of 7.45 dBi. Compared with the compact Vivaldi antennas reported in [18,19], the proposed design provides a wider fractional bandwidth while maintaining compact wavelength-normalised dimensions and a simple planar structure. Several reported designs achieve higher peak gains using dielectric lenses, metamaterials, corrugations, directors, metallic loading, or dielectric loading [27,28,32,34,37,38], generally at the expense of additional structural or fabrication complexity. In contrast, the proposed VTSARMT combines a radial-to-radial microstrip-to-slotline transition with an optimised matching transformer without requiring lenses, metamaterials, directors, parasitic elements, or multilayer structures.

The proposed antenna is experimentally characterised in terms of impedance matching, realised gain, radiation patterns, efficiency, phase, and group delay. Its rooftop integration is additionally investigated using stationary two-vehicle and three-vehicle CST configurations. These vehicle results provide a preliminary comparative assessment of rooftop integration and stationary directional-link behaviour rather than full-scale dynamic V2V-channel validation. In Table 2, VCA indicates whether vehicular communication applications were considered in the corresponding study.

## 5. Conclusions

This paper presents a compact ultra-wideband Vivaldi tapered slot antenna (VTSARMT) employing a radial-to-radial microstrip-to-slot transition together with a quarter-wave matching transformer to simultaneously enhance impedance bandwidth, realized gain, and compactness while maintaining a simple and low-cost structure. The fabricated prototype demonstrated a measured impedance bandwidth extending from 2.58 to 22.34 GHz for ∣S11∣<−10.61 dB, a maximum measured realized gain of 7.45 dBi, a measured radiation efficiency ranging from 47.7% to 94.08%, and an approximately stable group delay of 1 ns across most of the operating band. Compared with the measured reference VTSA, the proposed VTSARMT achieves a 16.67% footprint-area reduction and a 47.67% measured bandwidth improvement, while the maximum measured realized gain increases from 6.51 to 7.45 dBi.

To evaluate the effects of vehicle integration, the antenna was further investigated using stationary rooftop-mounted two-vehicle and three-vehicle configurations. The CST-based transmission-coefficient and received-power analyses were used to assess the selected stationary directional links at different antenna separations. The results demonstrate the broadband performance of the standalone fabricated antenna and provide a preliminary CST-based assessment of rooftop integration and stationary directional V2V links. Further experimental vehicle-mounted and system-level studies are required before extending these conclusions to dynamic V2V/V2I communication, localisation, vehicular sensing, or broader ITS applications.

## Figures and Tables

**Table 1:** Optimized parameters of VTSAR and VTSARMT compared to VTSA [8].

Parameters	VTSA [8]	VTSAR [51]	VTSARMT	
*r*	0.17			
*W_max_* (mm)	21.03			
*L_T_* (mm)	25			
*L_qw_* (mm)	5.7	4.6		
*W_min_* (mm)	0.3	0.3		
*radsl* (mm)	1.89	2		
*dis* (mm)	37.2	35		32
*W_f1_* (mm)	1.2	1.25		1.5
*W_f2_* (mm)	-	1.25		1.82
*W_m_* (mm)	-	1.25		2
*angsl* (°)	-	150		160
*angst* (°)	-	120		
*L_f_* (mm)	21.42	18.3		
*L_f1_*	-	-		6.6
*L_f2_*	-	-		5.09
*L_m_*	-			6.61
*Want* (mm)	42.9	39.6		36.6
*Lant* (mm)	29.28	28.6		28.6

**Table 2:** Comparison with recent wideband and ultra-wideband tapered-slot antennas.

Ref.	$\varepsilon_{r}$	Band (GHz)	FBW (%)	Gain (dBi)	Normalised Dimensions W/λ_g_ × L/λ_g_ × h/λ_g_	Compactness/Enhancement Technique	Feeding	VCA
VTSARMT	3.55	2.58–22.34	158.59	1.2–7.45	0.593 × 0.464 × 0.013	Radial-to-radial transition + matching transformer	M/S: radial/radial	Yes
VNSA [23]	3.55	2.63–12.33	129.68	2–5.9	0.383 × 0.244 × 0.013	Non-uniform slot profile	M/S: circular/circular	No
Antenna A [24]	3.55	3.16–14.34	127.77	1.9–6.0	0.382 × 0.246 × 0.016	VNSPA-based miniaturisation	M/S: circular/circular	No
Antenna B [24]	3.55	2.9–13.55	129.48	1.8–6.91	0.543 × 0.369 × 0.015	VNSPA-based miniaturisation	M/S: circular/circular	No
VTSA [9]	3.55	3.14–13.48	124.43	2.2–6.51	0.847 × 0.578 × 0.016	Compact transition modification	M/S: circular/circular	No
[37]	3.55	2–20	163.64	5.62–14.3	2.263 × 1.031 × 0.019	Embedded rhombus-shaped metamaterial array	Coaxial-to-microstrip	No
[39]	3.0	0.6–20	188.35	3.8–12.3	1.352 × 0.971 × 0.003	Dielectric lens + optimised slot	M/S: elliptical/elliptical	No
[18]	4.4	3.2–16.8	136.00	5–10.4	0.761 × 0.739 × 0.018	Sawtooth outer edge + inclined elliptical slots	M/S: circular/circular	No
[19]	3.5	2.32–13.75	142.25	3.1–7.9	0.521 × 0.521 × 0.011	Fan-shaped and stepped structures	M/S: circular/circular	No
[38]	4.2–4.8	3–8	90.91	7.2–8.75	2.256–2.412 × 1.969–2.105 × 0.016–0.018	Bottle-shaped slots + rectangular metal strips	M/S: rectangular/rectangular	No
[34]	4.3	1.1–8	151.65	4.9–10	0.624 × 0.418 × 0.012	Corrugations + director	M/S: radial/circular	No
[35]	2.2	2–11.79	141.99	0–7.9	0.396 × 0.455 × 0.016	Side slots + metamaterial	M/S: radial/circular	No
[47], 3 ports	NR	5–11	75.00	3–6.5	NR.: not reported	Corrugations	M/S: radial/circular	Yes
[33]	4.5	4.5–12	90.91	7.59–8.92	2.611 × 1.751 × 0.052	Corrugations + semi-circular structure	M/S: radial/circular	No
[46]	4.4	0.56–7.7	172.88	2.3–6.6	0.735 × 0.627 × 0.003	Two orthogonally crossed elements	Balun: radial/circular	Yes
[32]	2.2	0.7–12.7	179.10	1–3	0.087 × 0.087 × 0.003	Antipodal loading + slotting corrections	NR	No
[27]	3.48	1.34–18.5	172.98	Max. 10.7	0.417 × 0.438 × 0.006	Three metal patches	NR	No
[28]	3.38	3.5–16.5	130.00	5.3–13.1	0.740 × 0.610 × 0.033	Dielectric sheets	NR	No
[13]	3.36	2.6–20	153.98	1.5–11.3	1.113 × 0.795 × 0.008	Slot + air bridge between tapered flares	CPW/slot	No
[31]	2.2	3.3–13.9	123.26	2.4–9.9	0.620 × 0.473 × 0.016	HSR + transverse slots	M/S: radial/circular	No
[21]	4.4	0.36–4	166.97	6.2–7.5	0.302 × 0.290 × 0.002	Corrugations + resistors + short pin + patches	M/S: rectangular/square	No
[15]	1.6	3.55–12	108.68	3.57–7.54	0.914 × 0.872 × 0.024	Corrugations	M/S: radial/circular	No

## Data Availability

No new data were created or analyzed in this study. Data sharing is not applicable to this article.
